# Predicting rapid adaptation in time from adaptation in space: A 30-year field experiment in marine snails

**DOI:** 10.1126/sciadv.adp2102

**Published:** 2024-10-11

**Authors:** Diego Garcia Castillo, Nick Barton, Rui Faria, Jenny Larsson, Sean Stankowski, Roger Butlin, Kerstin Johannesson, Anja M. Westram

**Affiliations:** ^1^ Institute of Science and Technology Austria (ISTA), Klosterneuburg, Austria.; ^2^ CIBIO-InBIO, Centro de Investigação em Biodiversidade e Recursos Genéticos, InBIO, Laboratório Associado, Universidade do Porto, Vairão, Portugal.; ^3^ BIOPOLIS Program in Genomics, Biodiversity and Land Planning, CIBIO, Campus de Vairão, 4485-661 Vairão, Portugal.; ^4^ Department of Mathematical Sciences, Chalmers University of Technology and University of Gothenburg, Gothenburg, Sweden.; ^5^ Department of Ecology and Evolution, School of Life Sciences, University of Sussex, Falmer, Brighton BN1 9QG, UK.; ^6^ Ecology and Evolutionary Biology, School of Biosciences, University of Sheffield, Sheffield S10 2TN, UK.; ^7^ Department of Marine Sciences, Tjärnö Marine Laboratory, University of Gothenburg, 452 96 Strömstad, Sweden.; ^8^ Faculty of Biosciences and Aquaculture, Nord University, 8049 Bodø, Norway.

## Abstract

Predicting the outcomes of adaptation is a major goal of evolutionary biology. When temporal changes in the environment mirror spatial gradients, it opens up the potential for predicting the course of adaptive evolution over time based on patterns of spatial genetic and phenotypic variation. We assessed this approach in a 30-year transplant experiment in the intertidal snail *Littorina saxatilis*. In 1992, snails were transplanted from a predation-dominated environment to one dominated by wave action. On the basis of spatial patterns, we predicted transitions in shell size and morphology, allele frequencies at positions throughout the genome, and chromosomal rearrangement frequencies. Observed changes closely agreed with predictions and transformation was both dramatic and rapid. Hence, adaptation can be predicted from knowledge of the phenotypic and genetic variation among populations.

## Teaser

Spatial diversity patterns predict the evolution of both the genome and the shell of snails introduced to a new environment.

## INTRODUCTION

Populations can sometimes adapt rapidly to sudden environmental shifts, even within a few dozen generations (*1*, *2*). For many populations, rapid adaptation will be necessary to persist amid anthropogenic environmental changes (e.g., climate change, habitat fragmentation, and pollution) as well as after naturally occurring environmental shifts. However, we are far from being able to predict whether and how fast a population will adapt and which phenotypic and genetic changes will occur (*3*). These questions are of great interest in basic evolutionary biology (*4*, *5*). Adaptation relies on genetic variation, including both variation at individual base positions and larger structural variants (*6*, *7*). The latter include chromosomal inversions, which generate large gene blocks that are inherited together and can simultaneously affect multiple traits (*8*, *9*). Rapid adaptation particularly depends on variation already present within a species because time is not sufficient to accumulate new beneficial mutations unless population sizes are very large (*10*, *11*) and/or generation times are very short (*12*).

The reliance of rapid adaptation on preexisting variation suggests that it might be possible to predict future evolutionary change from knowledge of current variation (*13*, *14*). In particular, many temporal environmental changes, such as temperature increase, resemble a current pattern in space (e.g., a spatial temperature gradient). In this case, for a focal population experiencing an environmental change, adaptive evolution is likely to rely on genetic variation that has entered the population via past or ongoing gene flow from a population that has already adapted to a similar environment. Studies investigating phenotype-environment and genotype-environment associations often provide insights into spatial genetic variation. Can this knowledge on adaptive variation in space be used to predict how a population will respond over time after an environmental change? This principle is implicit in much conservation genetics work (*15*–*17*) but has rarely been explicitly tested (*18*, *19*). From a practical viewpoint, predictability would mean that population responses to environmental change can be anticipated and management efforts adjusted accordingly (*20*). In basic research, predictability provides a test of the current understanding of a system: For example, if loci contributing to divergence between environments in space have been identified correctly, they should respond in a predictable way to changing selection pressures in time.

**Fig. 1.**
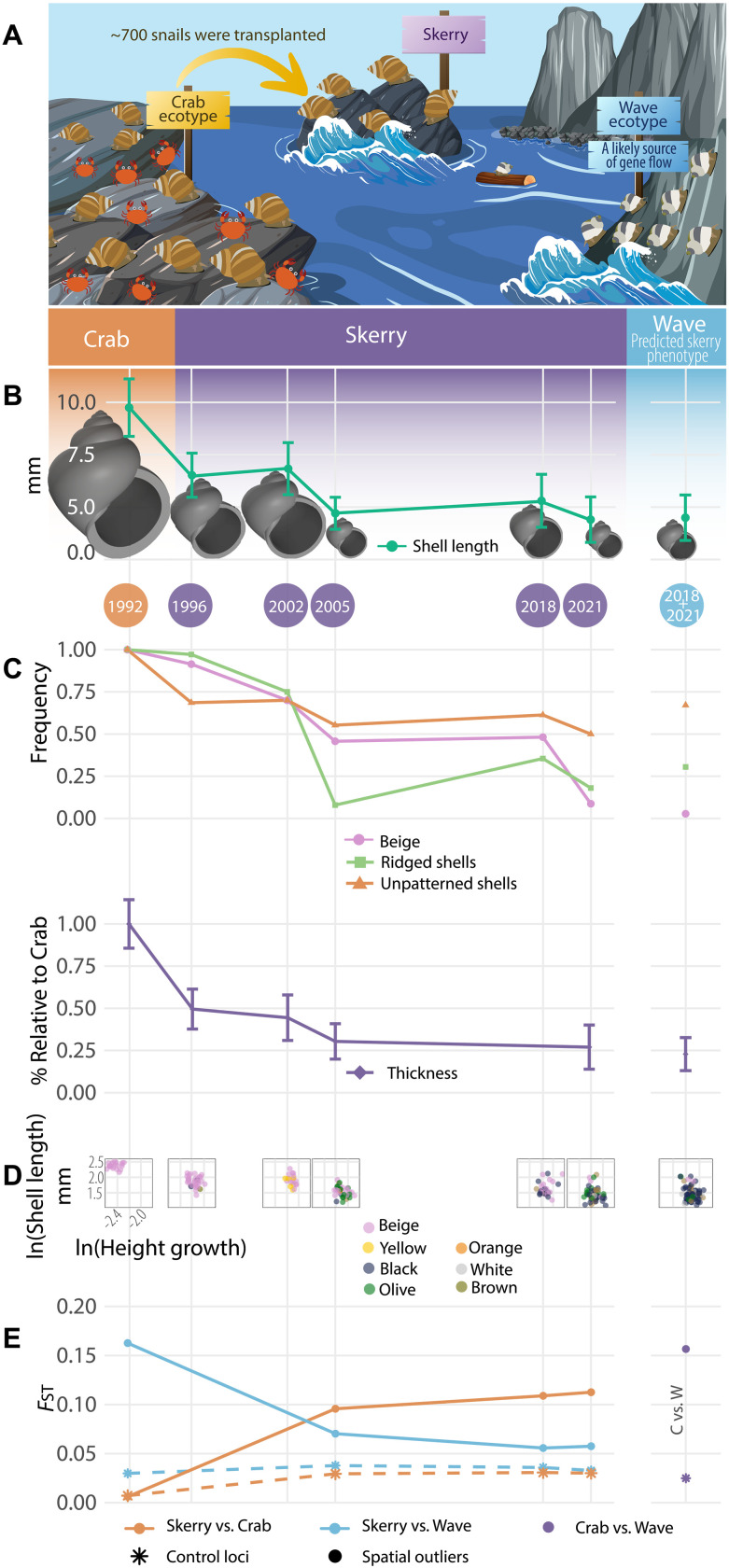
Divergence trajectory of the skerry population at the levels of phenotypes and loci in collinear genomic regions. Years correspond to sampling points in time. (**A**) Cartoon of the transplant experiment showing the donor Crab ecotype on the left side, the recipient skerry in the middle, and the neighboring Wave ecotype on the right side. Figure created using graphics from Vecteezy.com under free license. (**B**) Shell length and shape evolution toward the Wave ecotype on the skerry. (**C**) Evolution of shell color, patterning, ridging, and thickness in the skerry population toward the reference Wave ecotype. Thickness represents the average thickness relative to the average thickness of the transplanted population in 1992. (**D**) Scatter plots of two uncorrelated quantitative traits (shell length and height growth) on a log scale, and one qualitative trait (color) reveal no bimodalities in the skerry population. (**E**) Genetic differentiation of the skerry versus the reference populations based on control and spatial outlier SNP loci. The reduced-LD spatial outlier dataset was used.

The intertidal snail *Littorina saxatilis* is a model system in which divergent adaptation in space is exceptionally well-documented (*21*–*23*). Spatial variation and local adaptation to rocky shore environments are particularly obvious in the “Wave” and “Crab” ecotypes that have been intensively studied in Sweden, UK, and Spain. The ecotypes originated repeatedly in different locations (*24*), in response to the selective pressures of wave action (*25*) and crab predation (*26*) on wave-exposed rocks with low crab density, and sheltered crab-rich parts of shores, respectively (Fig. 1A) (*21*, *27*). Adaptive variation in space in this system has been studied on three levels. At the phenotypic level, the ecotypes differ in traits including size, shell shape, shell color, and behavior (*21*, *27*, *28*). The Wave ecotype is small, has a thin shell that often shows Wave-specific colors and patterns, a large and rounded aperture, and bold behavior, while the Crab ecotype is large, has a thick shell generally without patterns [but with a band patterning in Iberian Crab ecotype populations (*29*)], a relatively smaller and more elongated aperture, and wary behavior (Fig. 1B and fig. S1D). At the level of individual SNPs (single-nucleotide polymorphisms), highly differentiated loci likely contributing to adaptation or linked to adaptive loci are scattered across the whole genome (*23*, *30*). At the level of large chromosomal rearrangements, several inversions differ in frequency between ecotypes (*31*–*33*) and explain variation in divergent traits between ecotypes (*30*, *32*). These features all change over local contact zones between ecotypes, and most differences are paralleled over large geographic areas (*34*), strongly suggesting a role of divergent selection. Our main goal here is to test whether the observed spatial associations allow us to predict changes in time after an immediate environmental change.

While previous evolutionary experiments have demonstrated rapid change in predicted directions, they have limitations. Some have only looked at the end points of an adaptive process rather than a detailed trajectory (*2*, *35*, *36*), while others have been conducted in artificial environments (*37*–*40*). Our study is one of the few (*41*–*43*) to follow adaptation of a transplanted population in the wild from the beginning. Previous studies in stickleback (*42*), guppy (*41*), and salmon (*44*) primarily examined evolution in phenotypes and collinear regions. In our study, we show how natural selection reshapes phenotypes, structural variants (inversions), and collinear genes in predictable ways. In addition, disentangling the effects of selection and drift is still a challenge in evolutionary studies; we address this by explicitly inferring demographic parameters and then testing for selection against the expectation under neutrality (*43*, *44*). Our quantitative approach also allows for a detailed understanding of the history of our study population over the full three decades it has existed.

We assessed local adaptation in a 30-year transplant experiment on the Swedish west coast. In 1992, we collected ~700 Crab ecotype snails and relocated them to a nearby wave-exposed environment earlier occupied by a population of the Wave ecotype. This wave environment is a “skerry” (rocky islet, size approximately 1 m by 3 m), exposed to strong waves and with no evidence of crabs (Fig. 1A and fig. S1). The skerry had remained uninhabited by snails since a toxic algal bloom in 1988 killed them all (*45*). The skerry (current census size: ~1000 individuals) is located ~300 m away, across open sea, from the donor Crab ecotype population and ~160 m from the nearest Wave ecotype population (Supplementary Materials and Methods and fig. S1). Therefore, there are two potential sources of adaptive variation: standing genetic variation in the donor population (resulting, in part, from past gene flow from adjacent Wave populations on the same island) and posttransplant gene flow due to occasional migrants [e.g. rafted snails; see (*45*); the species is live-bearing and lacks pelagic dispersal] from the neighboring Wave population (or, less likely, elsewhere).

We predicted three levels of change in the skerry population. At the phenotypic level, we anticipated a transition from Crab ecotype to Wave ecotype morphology: The averages of quantitative traits (e.g., shell length and shell thickness) and the proportions of qualitative traits (e.g., shell color, patterning, and ridging) were expected to approach the values typically observed in the Wave ecotype present in the area. We formulated our prediction on the basis of the polygenic inheritance of phenotypes (*30*, *46*) that can reach Wave optima through different pathways (both genetic and plastic) and are often under strong selection in space (*47*). For SNPs, we predicted an allele frequency shift over time beyond the effect of drift and neutral gene flow in at least a subset of “spatial outliers” (SNPs associated with ecotype divergence in space in previous studies in the same geographical area; see Description of the sites, the translocation, and the subsequent sampling section in Materials and Methods) toward the frequencies observed in undisturbed Wave ecotype populations. For inversions, we predicted an increase in frequency of arrangements that are more common in Wave than in Crab ecotype populations. We predicted a tendency to fix arrangements that appear fixed in the Wave ecotype (*23*, *30*). We predicted non-fixation for inversions that are maintained polymorphic in the Wave ecotype, likely by balancing selection (*33*). Last, for both spatial outlier SNPs and inversions, we predicted a correlation between temporal (the start versus end of experiment) and spatial (Crab ecotype versus Wave ecotype) genetic differentiation. Overall, we expected the predictability to be higher for inversions than for SNPs because many inversions are likely to be under strong direct selection, while spatial outlier SNPs may often only be indirectly affected by selection.

## RESULTS

### Swift transformation of shell morphology and patterning confirms phenotypic predictions

To assess our predictions of a morphological shift from Crab ecotype to Wave ecotype following transplantation in 1992, we collected adult snails from the skerry and reference Crab and Wave ecotype populations during the season April to November in 1996, 2002, 2005, 2018, and 2021. As anticipated, the phenotypes of the transplanted snail population experienced multiple changes. In addition to a decrease in length, a shell reconstruction using six shape parameters (Fig. 1B, fig. S2, and Shell reconstruction section in Materials and Methods) showed that, after 30 years, snails from the skerry population had a broader aperture and less pointed tips than snails from the founding population, now being more similar to the Wave ecotype. Moreover, the beige color common in the Crab ecotype became rare over time, with the skerry population becoming color-polymorphic, similar to Wave ecotype populations (Fig. 1C). Simultaneously, the distinctively thick and ridged shells of the Crab ecotype were largely replaced by thinner and smoother shells, while nearly half of the population acquired shell patterning. Scatter plots depicting uncorrelated traits (Fig. 1D and fig. S4) show that this transition took place across all sampled individuals (Analysis of phenotypes section in Materials and Methods). Therefore, the change in the population average did not result from a bimodal distribution of phenotypes that coexisted on the skerry but rather from gradual changes across the entire population. Statistical tests confirmed significant differences between the 2021 skerry population and the Crab ecotype population (the donor population) for most qualitative and quantitative traits. Conversely, the differences between this skerry population and the nearby Wave population were either weak or nonsignificant, as predicted (tables S9 and S10).

Previous estimates of additive genetic variance and plastic effects of the environment for size and shell-shape traits allowed us to estimate the strength of selection required to explain the observed phenotypic changes in these quantitative traits (*30*). We used a model of Gaussian stabilizing selection toward an optimum defined by the phenotype of the Wave reference population (see Strength of selection based on phenotypes section in Materials and Methods). This model was assumed because it is consistent with the observed stability of phenotypes in Crab and Wave ecotype populations and predicts rapid initial change followed by a slower approach to the optimum. Assuming an initial plastic response in the transplanted population, the strength of stabilizing selection (*V*_s_/*V*_p_, the variance of the fitness function relative to the phenotypic variance) ranged from 3.8 to 25.3, depending on the trait, assuming two generations per year (Results of selection estimates based on phenotypes section in Supplementary text). These values are in the typical range for estimates from natural populations (*48*, *49*) and correspond to a fitness reduction for the Crab ecotype population, when first introduced to the skerry, between 16 and 80% (4 to 60% after the plastic change in phenotype). For the aperture position trait (*r*_0_), all of the change on the skerry could be accounted for by plasticity; for other traits, estimated plastic effects accounted for 15 to 50% of the change in phenotype on the skerry (table S2). Changes in these phenotypes were rapid and substantial because the transplanted population phenotypes were initially far from their optima on the skerry. Rates of change were high compared to evolutionary changes in other systems in the wild (*50*), induced by human disturbance (*51*), or suggested by theoretical models (*52*), especially for size and the growth parameter *g_w_* [rate of change >0.1 Haldanes (i.e., phenotypic SDs per generation) and extent of change >0.1 (absolute Darwin numerator, i.e., change as a proportion of the initial mean phenotype); see table S13].

### Multiyear genetic data confirm adaptive frequency shifts in candidate SNPs

To evaluate our predictions at the genetic level, we genotyped samples from different years (2005, 2018, and 2021) from the skerry population as well as from the donor Crab population (1992, 2018, and 2021) and the neighboring Wave ecotype population (2018 and 2021) (see SNP development and genotyping section in Materials and Methods). We included spatial outliers (292 SNPs, the full spatial outlier dataset) that showed high Crab-Wave differentiation in previous studies of ecotypes in the area (*23*, *34*), SNPs diagnostic for chromosomal rearrangements (225 SNPs) (*31*), regardless of whether they were outliers in previous studies or not, and control SNPs (565 SNPs) that lacked strong association with ecotype divergence in Sweden (*23*, *34*). To address potential bias from clustered linked loci, we performed window-based subsampling, resulting in a reduced-LD spatial outlier dataset of 56 SNPs. All spatial outliers and control loci are SNPs situated outside chromosomal inversions.

We predicted a temporal allele frequency shift in a subset of spatial outliers, approaching the allele frequencies observed in the Wave ecotype and surpassing neutral changes due to drift and gene flow from Wave. The allele frequencies at many control loci in the skerry population changed toward the frequencies observed in the Wave ecotype, suggesting neutral gene flow from the Wave population and/or hitchhiking with linked selected alleles: 59% of the control loci had shifted toward Wave in 2005, 63% in 2018, and 61% in 2021. For spatial outlier loci in the full spatial outlier dataset, the percentage that shifted toward Wave was larger, as predicted: 82, 87, and 89%, respectively (figs. S14 and S15). The trajectory of genetic differentiation ($F_{\mathrm{ST}}=\frac{H_{T}-H_{S}}{H_{T}}$, where *H*_T_ stands for total heterozygosity among both populations and *H*_S_ is within-population heterozygosity) also reflected the more pronounced shift of spatial outliers compared to the control loci (Fig. 1E; the reduced-LD dataset was used): The trajectory of spatial outliers indicates that from 2005 onward, the skerry population was not only highly divergent from the Crab ecotype but close to the Wave ecotype. Control loci, on the other hand, showed a weak directional trend in *F*_ST_. These results are also confirmed by a principal components analysis (PCA) (fig. S6).

**Fig. 2.**
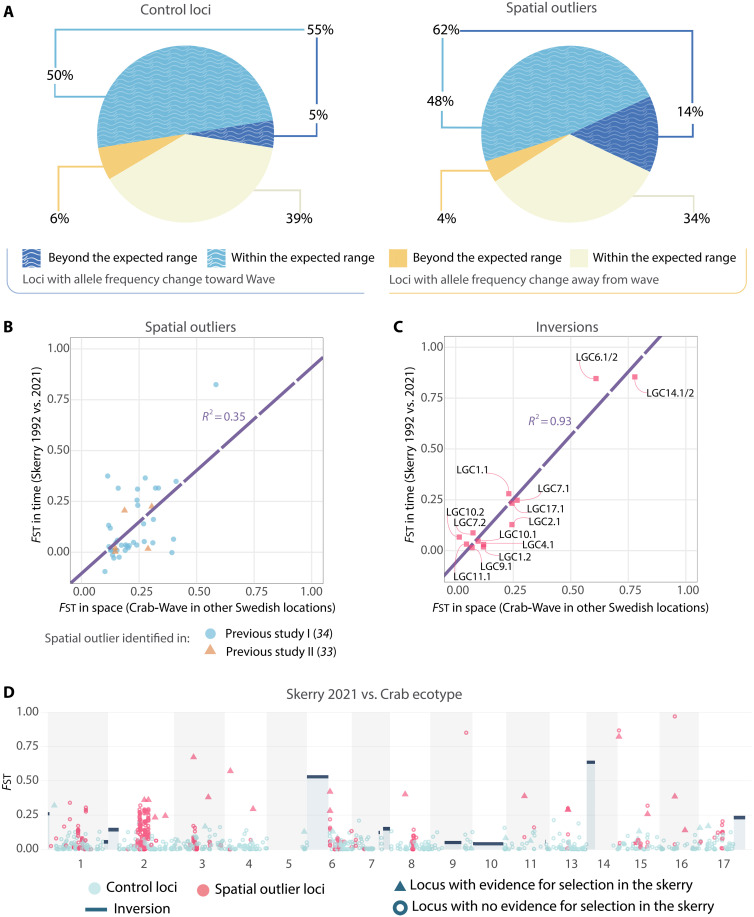
Genome-wide evidence of selection in the skerry. (**A**) A comparison to the expected 95% range of allele frequency changes without selection shows a strong allele frequency change toward Wave in a larger fraction of spatial outliers (reduced-LD spatial outlier dataset) than in control loci (blue wave-patterned). (**B** and **C**) Relationship between spatial and temporal differentiation for spatial outliers (reduced-LD spatial outlier dataset) and inversions. Positive temporal *F*_ST_ values indicate that the allele more common in Wave in space increased over time on the skerry, while negative values mean that the allele more common in Crab increased. The spatial *F*_ST_ reflects the average in nearby Crab-Wave contacts. The dashed lines represent the linear models describing the relationship (*R*^2^ = 0.35 and 0.93). (B) Spatial outlier SNPs identified in two previous studies: previous study I (*34*) (light blue) or previous study II (*33*) (orange). (C) Inversions. (**D**) Genomic distribution of *F*_ST_ in the skerry population versus the Crab ecotype population (2018 + 2021) for individual collinear SNPs (circles and triangles, the full spatial outlier dataset was used) and inversions (rectangular blue-gray fields with dark blue bars at the top). Triangles represent loci with evidence for selection toward Wave (allele frequency change beyond the expected range). The large number of spatial outliers in the center of LG2 is presumably due to the presence of a low-recombination region (*23*). Gaps in LG5 and LG14 correspond to the genetic map coordinates of putative inversions excluded from our analyses (*31*).

The fact that the allele frequency shift is more pronounced at spatial outlier loci than control loci is consistent with selection, although, alone, it does not provide sufficient evidence. This is because spatial outlier loci are on average more differentiated than control loci between the skerry starting population and the nearby Wave population. Therefore, neutral gene flow from a Wave population alone would already lead to a more pronounced shift for spatial outlier loci over time in the skerry population. To distinguish between these possibilities, we inferred the demographic history of the skerry population and compared the observed allele frequency changes to those expected under neutrality. On the basis of the allele frequencies of the control SNPs in the starting population (1992) and in the neighboring Wave population (2018 + 2021) as a potential source of gene flow, we found the growth rate, carrying capacity, migration rate, and number of generations per year that best predicted the allele frequency distribution observed in the skerry samples from 2005, 2018, and 2021 (Demographic inference section in Materials and Methods). The most likely estimate for migration from the neighboring Wave population was *M* = 1.63 diploid individuals per generation [support limits (1.39, 1.89); see table S5 for other parameters]. This relatively low number of migrants is reasonable considering that the species broods offspring internally and lacks pelagic larvae and that the skerry remained unoccupied by snails for 4 years after the toxic algal bloom (*45*). Furthermore, it is of the same order of magnitude as direct estimates of colonization of empty skerries in the area following the bloom (*45*). Starting with the allele frequencies observed in 1992, we simulated neutral evolution for each control and spatial outlier locus until 2021 (approximately 58 generations). For each SNP, we ran 1000 replicates, randomly drawing parameter combinations according to the likelihood surface of the demographic model. This allowed us to estimate the expected range of allele frequency changes from 1992 to 2021 without selection but including genetic drift, gene flow, sampling, and model uncertainty for each SNP (defined as the range spanning 95% of the simulated replicates) (fig. S13). While for both control loci and spatial outliers, the probability of being outside the expected range is relatively small (5 versus 14%), these deviations from the expected range are explained by systematic increases in Wave allele frequencies only for the spatial outliers, and this pattern is not a result of genomic clustering of spatial outliers (Fig. 2A, Results of the demographic inference section in Supplementary text). All spatial outliers that fell outside the expected range displayed significant allele frequency differences between the 2021 skerry population and the Crab ecotype population (Fisher’s exact test; table S11). In contrast, weaker or nonsignificant differences were observed with respect to the Wave ecotype population (Fisher’s exact test; table S11). In addition, in alignment with our prediction that selection influenced at least a subset of the spatial outliers, these generally shifted toward Wave more than expected without selection, even when those inside the expected range were also considered (62% of spatial outliers in the reduced-LD spatial outlier dataset showed a stronger shift than the median shift predicted without selection, compared to 55% in the control loci) (Fig. 2A). As selection is not expected to affect control loci directly, drift, gene flow, and hitchhiking effects are plausible reasons for the proportion of control loci that experienced an allele frequency shift toward Wave frequencies. Overall, both the allele frequency changes over time, and the extent of deviation from the expected range confirm our prediction of a more pronounced shift toward Wave frequencies in spatial outliers than in control loci.

As predicted, spatial outliers as well as inversions that were more differentiated between Crab and Wave ecotypes in other locations in Sweden tended to show a greater allele frequency change over time in the skerry; however, the relationship was noisier for individual SNPs than for inversions (Spearman’s rho = 0.47, *P* = 0.0015 in the reduced-LD spatial outlier dataset versus Spearman’s rho = 0.76, *P* = 0.0036; Figs. 2, B and C). Loci potentially affected by selection are distributed widely along the genome (Fig. 2D and fig. S16). We found no evidence for over- or underrepresentation of temporal outliers in specific linkage groups (LG) compared to the chance expectation (loci with evidence for selection chosen randomly from our full set of spatial outliers). The only exception was that LG2, which contains a large cluster of spatial outliers [presumably reflecting a low recombination region (*23*)], showed fewer loci with evidence of selection on the skerry (red triangles) than expected (fig. S17).

### Natural selection favors specific inversion arrangements as predicted

**Fig. 3.**
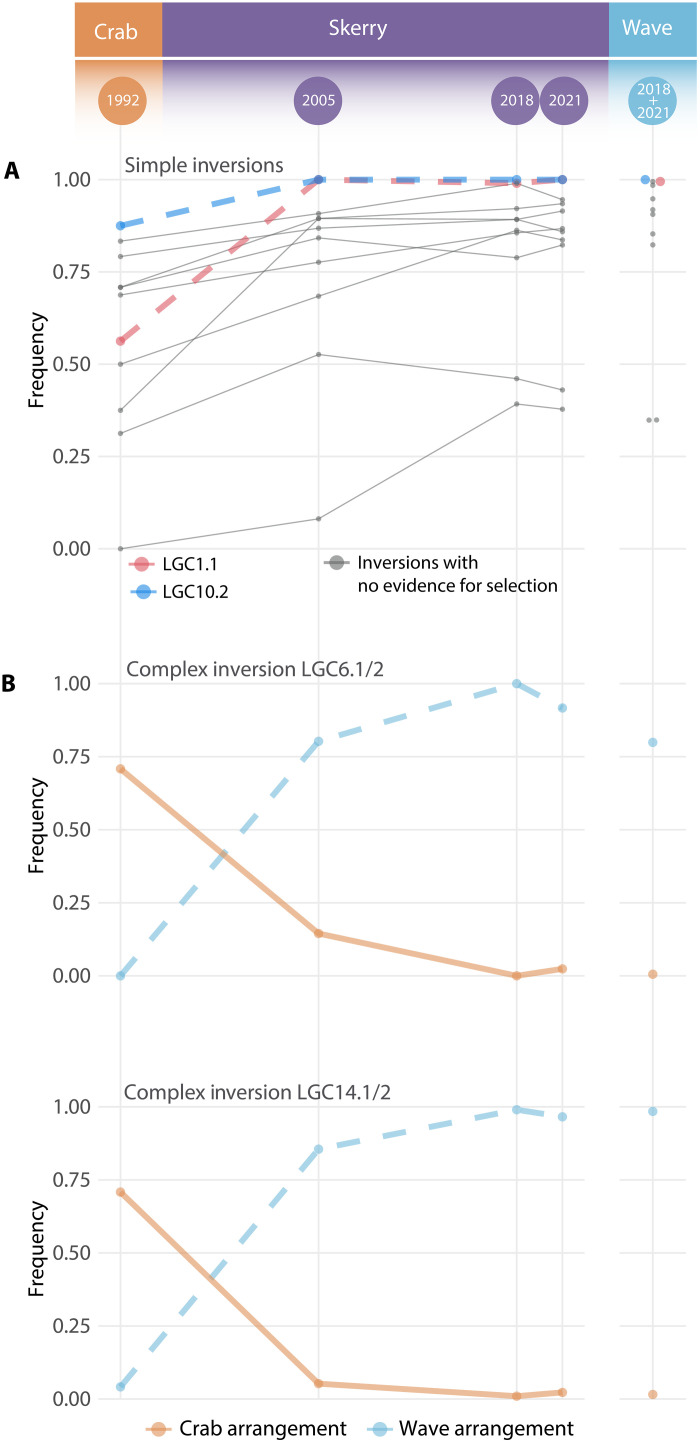
Trajectories of the Wave arrangement for polymorphic inversions. Gray lines indicate trajectories of arrangements in the skerry population with frequency changes between 1992 and 2021 within the range expected without selection. The trajectories of arrangements that experienced frequency changes beyond the expected range are shown in color. (**A**) The trajectories of simple inversions show arrangements of two inversions (LGC1.1 and LGC10.2) that changed in frequency more than expected by drift and gene flow alone. (**B**) The Wave arrangement in complex inversions (dashed line) on two linkage groups (LG6 and LG14) increased from rare to near fixation, while the Crab arrangement (solid line) became rare.

We predicted that inversion arrangements more prevalent in the Wave ecotype than in the Crab ecotype would increase in frequency and, in some cases, fix in the skerry population, reflecting the frequencies that are typical in Wave populations (*31*). To test this, we tracked the 30-year trajectory of “Wave arrangements” in two inversion types. Trends in both “simple” and “complex” inversions, with two and three alternative arrangements, respectively, confirm our prediction (Fig. 3; Inversion frequency estimation section in Materials and Methods). While the karyotype frequencies in all 17 inversions differed significantly in the 2021 skerry population compared to the Crab ecotype population, weaker or nonsignificant differences were observed compared to the Wave ecotype population (Fisher’s exact test; table S12). Simulations of neutral expectations (see Simulation of inversion trajectories within the expected range of frequencies without selection section in Materials and Methods; figs. S12 and S13C) show that arrangement frequency shifts required selection in four cases (simple inversions LGC1.1 and LGC10.2, and both complex inversions, LGC6.1/2 and LGC14.1/2). The two complex inversions, in particular, are strongly associated with Crab and Wave ecotype divergence and influence adaptive traits (*23*, *30*). For example, LGC6.1/2 influences size-related measures (weight, thickness, and shell length), and LGC14.1/2 might be associated with weight, but evidence so far is inconclusive (*30*, *32*). As we also predicted, inversions that are highly genetically differentiated (*F*_ST_) between ecotypes in space exhibited the greatest differentiation between 1992 and 2021 within the skerry population (Fig. 2C). Moreover, the inversions showed growing *F*_ST_ over time in the skerry population with respect to Crab, comparable to the differentiation observed in spatial outliers (Fig. 2D and fig. S16).

## DISCUSSION

Although some studies suggest that evolution is inherently unpredictable because of the interplay of mutational randomness, phenotypic complexity, and environmental dynamics (*53*, *54*), investigations involving different species have revealed patterns of change over time that align with spatial predictions. Such studies include stickleback (*39*, *40*, *42*), guppy fish (*43*), and mouse (*55*). Expanding on this work, we tested the predictability of adaptive evolution at three different levels of variation based on spatial patterns—phenotype, SNP genotype, and chromosomal inversion. In barely a decade after the introduction, a transplanted population of Crab ecotype adapted to a new environment that closely resembles that of the Wave ecotype.

In our study, patterns of transformation on the three studied levels (i.e., phenotypes, collinear SNPs, and inversions) match our predictions. However, predictability is only high for phenotypes and for strongly selected inversions. Phenotypically, the skerry reached a Wave ecotype end point, as predicted, through the contribution of both the spread of adapted alleles over several generations and plastic responses to the new environment immediately following transplant. The latter represents a major adaptive mechanism that enables organisms to persist in an altered environment, while allele frequencies in the population adapt to a new optimum (*56*). At the inversion level, the Wave arrangement in all cases reached frequencies similar to those in a Wave ecotype population, with statistical evidence for selection in four cases. This is in line with their widely accepted role in suppressing recombination between alleles beneficial in a specific genetic background or environment (*57*, *58*).

Predictability is lower for our studied set of collinear loci. In accordance with our prediction, spatial outliers experienced greater allele frequency changes over time, beyond chance effects, compared to control loci. However, only a small fraction of the spatial outliers showed clear evidence of selection, and we were not able to predict this specific subset of loci. Multiple, nonmutually exclusive explanations exist. First, some loci detected as spatial outliers in other locations may not be under selection on the skerry due to environmental discrepancies between different Wave habitats. For example, the skerry is flatter than previously studied Wave ecotype locations and only represented by low-shore habitat, while adaptation to vertical shore gradients has been observed in other Wave ecotype locations (*34*). Second, many of our spatial outliers may be linked to targets of selection rather than being under selection themselves; in this case, responses to selection depend on the linkage disequilibrium in the studied population. In line with this observation, allele frequencies at some collinear loci did not exhibit the expected pattern of change under a constant selection coefficient (inferred by maximum likelihood estimation; fig. S19). At these SNPs, while the frequency of the Wave allele initially rose swiftly at the onset of the experiment, it subsequently leveled off (fig. S19). This pattern suggests that the genotyped marker gradually dissociated from a selected allele over time. Third, except for color traits that are coded by single genes (*59*), many of the adaptive traits are likely to be highly polygenic, e.g., shell size and shape (*32*, *46*), and the same phenotypic optimum can potentially be reached via different genetic routes (*30*). Therefore, it is plausible that the evolution of the Wave phenotype was possible via changes at a subset of the loci studied here, together with changes at loci not included in this study. Thus, more information on collinear targets of selection may improve predictability.

Disentangling the effects of demographic history and natural selection is challenging (*60*). Demographic modeling played a critical role in reconstructing the genetic history of the introduced population and assessing the accuracy of predictions. The robustness of our model stems from incorporating known information about the skerry, the donor population, and a potential gene flow source. Nevertheless, limitations may still exist. For example, if alleles introduced by migration experience positive selection and large blocks of migrant (Wave) background hitchhike along, we might overestimate the number of migrants. However, this does not affect our result that spatial outliers and inversions shift more toward Wave than expected without selection. In addition, we did not find evidence that hitchhiking extends over a larger region of the chromosomes (fig. S18).

Much recent interest in adaptation genomics has revolved around the origin of adaptive variation. We were not able to trace the origins of adaptive alleles directly with our SNP genotyping data. However, using simulations, we could estimate the proportion of Wave allele copies on the skerry at the end of the experiment that originated by gene flow from Wave versus from standing genetic variation in the Crab donor (fig. S19). This proportion can also be approximated using basic population genetic principles (see Mathematical approximation to the origin of the adapted alleles in Supplementary text and fig. S19). The results indicate that both sources of genetic variation are likely to have played a role (fig. S19).

In summary, our study presents a clear example of rapid evolution that is highly predictable in time based on spatial patterns. In the Anthropocene, a key question is whether such results are transferrable to situations with environmental changes that result from human activities, e.g., climate change, industrial fishing, habitat fragmentation, and alien species invasions (*61*). Future experiments could integrate variables associated with these changes (e.g., temperature, precipitation, and pollution). In our study, rapid adaptation was possible because the introduced population had access to a substantial amount of relevant genetic variation that had evolved in response to a similar environmental challenge across a diverse population. Genetic variation was available in the form of standing genetic variation in the founder population as well as the likely introgression of alleles carried by migrants from the reference Wave ecotype. Likewise, the availability of genetic variation in the area (i.e., in the Swedish archipelago) enabled us to formulate and confirm our predictions of phenotypic and genetic changes in a directional trend toward the observed values in the reference Wave ecotype. Similar conditions might apply where populations need to adapt to rapid anthropogenic change that mimics spatial gradients, such as temperature and other changes with altitude, provided that the populations are well-connected. In contrast, where populations need to adapt to anthropogenic changes, such as pollution or novel temperature extremes, available genetic variation may not be sufficient. This is because no population of the same species has experienced these novel environments in the recent past. In this case, adaptation would require de novo mutation, which is less predictable (because there are no adequate populations to base predictions on) and, more importantly, might often not be possible at all because beneficial mutations accumulate too slowly. The population might decline and eventually go extinct. Our results highlight the importance of maintaining populations across a variety of different environments, preserving the genetic variation needed to fuel future adaptation, especially in the context of climate change (*52*).

## MATERIALS AND METHODS

### Description of the sites, the translocation, and the subsequent sampling

The study area is part of the Kosterhavet National Park on the Swedish west coast. It is an archipelago of hundreds of small islands and rocks (fig. S1). The skerry is a tiny isolated rock: 1 mm by 3 m wide and 0.5 m high above mean water level (58.84128N, 11.05111E). It is separated by 160 m of 5- to 20-m-deep water from the nearest island. The area has only a very restricted tidal range (max 0.35 m), and the rock is only occasionally completely submerged. In 1985, a population of about 1000 Wave ecotype *L. saxatilis* inhabited the skerry. In May 1988, this population was wiped out by a unique bloom of toxic microalgae (*Chrysochromulina polylepis*) (*45*), and visits in June and October of the same year and in summer 1989 confirmed that the snail population was completely extinct on the skerry.

A visit in May 1992 again showed that recolonization had not taken place, and one of us (K.J.) decided to translocate Crab ecotype snails from a nearby donor population. This donor site (58.84397N, 11.05279E) is a boulder shore 320 m away from the skerry. It is densely populated by *L. saxatilis* of Crab ecotype over 100 m of shore, which roughly means >20,000 adult snails. This Crab ecotype population is flanked by snail populations of the Wave ecotype on both sides, and gene flow is likely to contribute to the presence of low frequencies of Wave-adapted alleles in the standing genetic variation of the Crab population. In this respect, the donor population was typical of Crab ecotype populations on the west coast of Sweden. On 30 May 1992, approximately 600 to 800 adult Crab ecotype snails were moved from the donor site to the skerry. A subsample of Crab ecotype from the donor site was stored as a reference. One month later, only 50 of the translocated snails could be observed on the skerry, but, most likely, approximately several hundred 0.5 mm of juveniles (invisible to the naked eye) were already released on the skerry. (The species broods offspring internally, and females are fertile year-round and release on average one to two juveniles per day).

Sampling of adult snails from the skerry was undertaken in 1996, 2002, 2005, 2018, and 2021, during 2018 and 2021 under a permit from the Regional County Board regulating scientific studies in the National Park inaugurated in September 2009 (permit number 521-3544-2018). Before September 2009, no special permits were needed. On each occasion, only a small proportion of the population was sampled, typically about 30 to 35 snails. New samples were also taken from the donor site in 2018 and 2021 and from a Wave ecotype reference population (58.84066N, 11.04794E) 160 m west of the skerry. The reference site represents the closest Wave ecotype population to the skerry and is one of several nearby Wave populations from which migration of single snails could have taken place. Following the extinction of all snails on intertidal skerries in the area, after 4 years, 27% of the skerries had received migrants, and in 12% of the skerries, population sizes were restored (*45*). While skerries only a few meters from islands had higher migration rates than more remote skerries (like the experimental skerry of the current study), this still indicates a potential for a small number of migrants to arrive on the experimental skerry. The arrival of migrants between 1992 and 2021 might have occurred by rafting from surrounding populations or as stowaways after passing through the digestive system of marine birds as vectors (*62*), in addition to the snails translocated from the donor population in 1992.

### Phenotyping

Phenotyping of shell traits included shell length, shell thickness (mean of three measurements per snail), shell color and pattern, shell ornamentation, and size-independent parameters for shell shape. The shell shape was quantified and analyzed using the set of growth-related parameters from a previous study (*27*), which were extracted from photos using the software ShellShaper. The same measurements were made on all samples, with the exception of shell thickness that was not measured for 2018 samples. Shells were categorized into seven color groups: beige, yellow, black, olive, orange, white, and brown. The classification as patterned (tessellated) or ridged was determined by the presence or absence of ornamentations (such as stripes) and striations on the shell, respectively. Datasets are available online on https://github.com/fernandoGarcia21/littorina_saxatilis_skerry/tree/main/Data.

### SNP development and genotyping

We developed a panel of SNPs and genotyped our samples using a targeted genotype-by-sequencing service provided by LGC Bioresearch Technologies. We first identified a large number of SNPs (20,000) based on previous genomic studies of Crab and Wave ecotypes in *L. saxatilis.* These included (i) “control” SNPs that show no prior evidence for selection between the ecotypes across Europe (*34*), (ii) SNPs that show selection based on cline analysis across a single Crab-Wave transition in Sweden (ANG; Ängklåvebukten) (*33*), (iii) SNPs that show evidence for selection in a Baypass outlier scan performed on multiple Crab-Wave contrasts across Europe (*34*), (iv) SNPs that show high differentiation (based on *F*_ST_) in one or more of five Crab-Wave contrasts in the Koster marine park in Sweden [where the experimental skerry is located (*34*)], and (v) SNPs that are diagnostic for chromosomal inversions polymorphic across the Crab-Wave transition at ANG (*23*, *31*, *33*).

From this large set of possible SNPs, we then selected 5000 for an initial genotyping trial. For the control SNPs, we chose one marker from each of 1000 map positions [according to a previously published genome and genetic map (*33*)], and a minor allele frequency greater than 0.1. We chose 1161 Baypass outliers, biased toward markers with stronger evidence of selection; most from Bayes factor categories 2 (Bayes factor >15) or 3 (Bayes factor >20). We also chose 16 diagnostic SNPs for each inversion, selecting those with the most power to distinguish the alternative arrangements. For the other categories, all potential SNPs were tested.

SNPs from this trial were carried forward if (i) they were successfully genotyped in at least 80% of individuals (with calls requiring at least eight reads) and (ii) they were biallelic, single position variants. In the second trial, 24 individuals were genotyped using a different annealing temperature to improve probe hybridization. In addition to the above criteria, we only retained SNPs where the two alleles in heterozygotes were at a frequency between 0.3 and 0.7. The final selection of SNPs was chosen to maximize spread across the genome, including representation of collinear and inverted regions.

For all analyses shown here, for SNPs in categories (iii) and (iv), we used only those that specifically showed evidence for selection in the Swedish archipelago this study was performed in (i.e., loci that were above the 95% quantile of the *F*_ST_ distribution in the locations Arsklövet, Saltö, Ramsö, and Jutholmen). For category (i), we discarded SNPs above the 95% *F*_ST_ quantile in the Swedish locations. We also excluded all SNPs in LG12, a region that has been associated with sex determination (*30*), and most SNPs in LG5, which might be part of a putative inversion (*31*).

The SNPs retained in categories (ii), (iii), and (iv), with evidence for divergent selection between Crab and Wave ecotypes, constituted our set of spatial outliers. To address the possible bias that spatial outliers in strong LD can introduce to the results, we subsampled this category of loci as follows: There were, in total, 292 SNPs in the full spatial outlier dataset. We subsampled to one random SNP per 1-cM window. For LG2, we removed all SNPs between 36 and 60 cM, where there is a large cluster of spatial outliers. After these subsampling steps, 56 spatial outlier SNPs across the collinear genome remained and we refer to these as the reduced-LD spatial outlier dataset.

All aspects of the DNA extractions and genotyping were performed by LGC Bioresearch Technologies. The final counts of SNPs retained across each category in this study are provided in table S1. Datasets available online on Zenodo (*63*) and Github at https://github.com/fernandoGarcia21/littorina_saxatilis_skerry/tree/main/Data.

### Data filtering

We used vcftools to filter the SNP dataset as follows; minimum SNP quality of 40 (--minQ 40), exclude indels (--remove-indels), exclude all genotypes with a quality below 20 (--minGQ 20), keep only genotypes with at least 10 reads (--minDP 10), keep only biallelic SNPs (--min-alleles 2 --max-alleles 2), and keep only sites with a minor allele count of 5 (--mac 5). Additional filters were applied in R. To estimate allele frequencies in collinear regions (control and spatial outlier loci), we included only SNPs with data for at least five individuals within a population and a year. For PCA on collinear loci, we excluded individuals with more than 5% missing genotypes and SNPs with more than 5% missing data within one population and a year. For the analysis of inversions, we excluded individuals with more than 20% missing genotypes, as well as SNPs with more than 20% missing data within one population and year. For PCA analysis of both collinear loci and inversions, we replaced the missing genotypes with the most common genotype within each population and year.

### Shell reconstruction

The description of shell shape is based on a logarithmic helicospiral growth model developed specifically to capture the shape variability present in the shells of different ecotypes of *L. saxatilis* (*27*). Parameter values representing shape and growth were inferred from two-dimensional (2D) images of shells in a standardized orientation and used as quantitative morphological measurements. Since these parameters represent an approximation of the shell construction process, they give biologically relevant shape descriptions and enough information to generate 3D models of the shells.

In this analysis, we used six parameters to quantify the shell shape (fig. S2), which have previously been found to correlate with the Crab-Wave ecotype differentiation in *L. saxatilis*. The parameter values were obtained using the program ShellShaper (https://github.com/jslarsson/ShellShaper), which infers the values from user input in the form of reference points and curves placed onto a standard orientation shell image. The program also generates 3D models from the inferred set of parameter values for each shell or for the average shell in a set of samples, which we are using to visualize the morphological changes over time.

### Strength of selection based on phenotypes

The strength of selection acting on phenotypic traits was estimated for shell length and for five components of shell shape variation (*g_w_*, *g_h_*, *a*_0_, *r*_0_, and *c*; width growth, height growth, aperture radius, aperture position, and aperture shape, respectively) based on a previously developed growth model (see the previous section) (*27*). Growth parameters *a*_0_ and *r*_0_ were expressed relative to shell length. The parameter *g_w_* was analyzed without transformation as in previous studies (*30*), and *g_h_*, *r*_0_, and shell length were log transformed.

For each phenotypic variable, we assumed stabilizing selection around an optimum phenotype, *O*

$$w=e^{-\frac{{\left(x-O\right)}^{2}}{2V_{s}}}$$

where *w* is the fitness of an individual of phenotype *x*, and *V*_s_ is the width of the fitness kernel. The expected change in mean phenotype in one generation is then

$$∆x=\left(O-\bar{x}\right)\frac{V_{g}}{\left(V_{s}+V_{p}\right)}$$

[Charlesworth and Charlesworth (*48*), p.186], where *V*_g_ is the additive genetic variance in the trait and *V*_p_ is the phenotypic variance. Offspring of individuals introduced to the skerry would have had Crab genotypes, but their phenotypes would have been influenced by plastic responses to the skerry environment. Therefore, our expectation for the phenotypic mean of the starting population on the skerry was ${\bar{x}}_{\text{crab}}+p$, where *p* represents the plastic effect.

To fit this model to the available phenotypic data, we assumed that the optimum on the skerry is the same as the optimum phenotype in the environment of the Wave reference population and can, therefore, be estimated by the phenotypes of individuals sampled from that population. We used previously analyzed data from three nearby transects (*30*) to provide estimates of *p* and *V*_g_. Specifically, environmental effect estimates (*30*) were used to predict the change in phenotype for a Crab individual placed in the Wave habitat in the lower half of the shore height range. The mean of the estimates for the three sites and the variance among sites were used to provide a Gaussian prior distribution for *p*. Similarly, a prior for *V*_g_ was set using the background additive genetic variance and inversion effects estimates from the previous study (*30*). We used either estimates from the Crab populations only (*V*_g_-crab) or from the whole transects (*V*_g_-site) in separate analyses to bound the likely range of genetic variation available on the skerry, which derives from the Crab introduction plus input from migrant Wave individuals. We assumed either one or two generations per year: The average generation time probably lies between these two values. Estimates from all combinations are provided in table S2.

The model was then fitted using R-Stan version 2.21.7 (*64*). All samples from the Crab donor population were combined to estimate the starting mean phenotype, and both samples from the Wave reference were combined to estimate the Wave optimum phenotype. Predicted phenotypes were fitted to the skerry samples. The phenotypic variance, *V*_p_, was assumed to be constant across all samples, with a Gaussian distribution. We checked this assumption by comparing samples using Levene’s test and found no significant departure from homogeneity of variances with the scaling used. We fitted the ratio *V*_s_/*V*_p_ since this is comparable across phenotypes, and we set a flat prior from 0 to 50 based on the values reported for natural populations (*48*, *49*). We also estimated the expected fitness of the mean Crab phenotype in the Wave environment. We used four chains of 3000 iterations, discarding the first 1000 iterations in each case as burn-in.

### Analysis of phenotypes

The general pattern we observed in the phenotypic traits, both quantitative (such as shape parameters, length, and thickness) and qualitative (including ridging, color, and patterning), indicates that the skerry population evolved a morphology similar to that of the Wave ecotype. However, we wondered how this transition from a Crab-like morphology to a Wave-like morphology occurred in the early stages. For instance, did this transition happen smoothly for the entire population, or was there a period during which more than one class of phenotype coexisted on the skerry, with some individuals exhibiting a more Crab-like appearance and others displaying a more Wave-like one?

We first identified the most informative quantitative traits: pairs of traits that are negatively correlated between the ecotypes (all data) but uncorrelated within an ecotype (fig. S3A), for example, the pair width growth and average thickness. Second, among the candidate informative traits, we chose only those traits where there is a relatively large difference between the Crab and Wave ecotypes. Our criterion was that we should not observe individuals of one ecotype within the interquartile range of the other ecotype (fig. S3B). Thus, the most informative quantitative traits are as follows: thickness, shell length, height growth, and width growth.

We then plotted pairs of uncorrelated traits in a scatter plot colored by the three qualitative traits (fig. S4). We did not observe strong evidence for bimodalities in the scatter plots. Rather, the phenotypic transition of the skerry population occurred in the whole population. For a better visualization of the changing distribution of phenotypes over the years, we generated bar plots of the quantitative traits including qualitative data (fig. S5). The distribution of quantitative traits tends to shift toward Wave ecotype faster than qualitative traits. For example, in 1996, the distribution of the shell length was shifted to the left, but the frequency of ridged shells and the color remained more or less the same as in the founder population.

### Demographic inference

We inferred the demographic history of the skerry population using maximum likelihood. For that, we needed to calculate the probability of obtaining the observed allele frequencies in samples from the skerry, given a set of demographic parameters. The set of model parameter values that maximizes this probability represents the most likely history of the population.

Our main goals were as follows: first, to test whether we can exclude gene flow from the nearby Wave population, in which case adaptive change on the skerry must result from selection on standing genetic variation from the Crab donor; second, to obtain the expectations for allele frequency changes under neutrality to test whether spatial outlier loci deviate significantly from this neutral model. All analyses were run in R version 4.2.1, and for some parameter combinations, an independent check was run in Mathematica v. 12.3 (Supplementary Materials file Skerry interpolation 9.23 v2.nb).

#### Data

As we aimed to infer the history of the skerry population, the demographic inference focused on control SNPs. To reduce the effects of linkage, we only used the first control SNP from each contig (*n* = 438 SNPs). For the skerry, we had information from four sampling times: 1992 (when a sample from the Crab donor population was taken at the same time as collecting the donor individuals for the skerry), 2005, 2018, and 2021. The four allele frequency estimates on the skerry were the observed data whose probability we aimed to maximize. In addition, we needed information about allele frequencies in the Wave reference population to infer the extent of gene flow. As we assumed the Wave population to be stable over time (see fig. S6, PCA on collinear loci), we merged the 2018 and 2021 samples from the Wave population for this analysis.

To avoid missing data, we determined the minimum number of (diploid) individuals with data per SNP for each sample (Skerry 1992, 2005, 2018 or 2021; Wave 2018 + 2021) and then subsampled all other SNPs to that sample size. This led to the sample sizes shown in table S3.

#### Model

We assumed a simple model of population growth with gene flow (fig. S7 and table S4). The model assumes that the skerry population was founded with *N*_0_ haploid genomes (i.e., $\frac{N_{0}}{2}$ diploid individuals) sampled from the Crab donor population. We then assumed logistic population growth at rate *r* with carrying capacity *K* haploid genomes, where the population size at time *T* is

$$N_{T}=\frac{K}{1+\left(\frac{K-N_{0}}{N_{0}}\right)\;\text{exp}\left(-\mathrm{rT}\right)}$$

There is unidirectional gene flow of *M* haploid migrants per generation from the Wave population. We assumed nonoverlapping generations, with *f* generations per year, as the exact generation time of *L. saxatilis* for this population is unknown. Sampling therefore took place at the start of the experiment (1992), after 13*f* generations (2005), after another 13*f* generations (2018), and after an additional 3*f* generations (2021). Given that there cannot be fractions of generations in our model, each sampling generation was rounded to the nearest integer, and the total duration of the experiment was *round*(13*f*) + *round*(13*f*) + *round*(3*f*) generations.

The parameter values tested are described in table S4. We included a large range for *N*_0_ because prior information about the starting effective population size is limited despite the known number of transferred individuals. On the one hand, it is likely that a considerable proportion of the transferred individuals did not attach to the rock and were immediately lost at the start of the experiment; on the other hand, because adult *L. saxatilis* females typically carry offspring from multiple fathers in their brood pouch, the effective starting population might have been larger than the number of adults. We also included a wide range of growth rates as little is known about the speed of population growth. The maximum carrying capacity *K* included was based on the fact that ~2000 snails were estimated to live on the skerry in 2014 when the population was well-established. Numbers of (haploid) migrants were capped at eight per generation because the skerry remained empty for 4 years after the algal bloom, indicating that migration rates are unlikely to exceed a couple of migrants per year on average. The tested values of generation factor *f* were based on generation times of *L. saxatilis* in the laboratory at similar temperatures (*65*).

#### Inference

To infer the most likely combination of parameters, we must calculate the probability of seeing the observed allele counts in the skerry sample for each combination of parameters.

The observed allele counts on the skerry at the different sampling times for a given SNP can be described by a vector **k** = {*k*_1992_, *k*_2005_, *k*_2018_, *k*_2021_}. For each sampling time, the probability of sampling *k_T_* depends on the real allele count at that time, *i_T_*, in the population, and on the probability that the population has actually evolved to this count. The probability of sampling the vector **k** is therefore

$$p\left(k\right)=\sum \psi_{92}s_{92}M_{92\to 05}s_{05}M_{05\to 18}s_{18}M_{18\to 21}s_{21}$$

where the sum is over all possible combinations of *i_T_*. This sum occurs because each *i_T_* is unknown and we therefore need to sum over all possible allele counts on the skerry.

**ψ**_92_ is the prior distribution of allele counts in the starting population on the skerry. We assume a uniform prior, i.e., $\psi_{x}=\frac{1}{N_{0}+1}$ for each possible allele count *x*.

*s_T_* is the probability of sampling *k_T_* copies from the *i_T_* copies in the population at time *T*. This is a binomial sampling probability where the parameters are the sample size at sampling time *T* and *i_T_*.

*M*_*T_a_*→*T_b_*_ is the probability of evolving from *i_T_a__* to *i_T_b__* copies in the interval from *T_a_* to *T_b_*. This is a transition matrix describing the evolutionary process on the skerry. This matrix is calculated as follows. Following basic population genetic principles, the expected allele count *i* on the skerry after a single generation of migration from the reference Wave population is

$$i_{T_{\exp}}=\frac{i_{T-1}}{N_{T-1}}\left(N_{T-1}-M\right)+p_{\text{Wave}}M$$

From this, the probability of *i_T_* copies in the population at time *T* can be calculated from a binomial distribution with parameters *N_T_* and *i_T_exp__* (the binomial sampling here represents the process of genetic drift). In the full matrix, the rows reflect all possible *i*_*T*−1_, i.e., all possible allele counts in generation *T*−1 (0,1, …*N*_*T*−1_), the columns reflect all possible allele counts in generation *T* (0,1, …*N_T_*), and each cell gives the probability of evolving from *i*_*T*−1_ to *i_T_* copies.

To obtain the negative log-likelihood for any parameter combination, we calculated *p*(*k*) as described above for each SNP, took the negative log, and summed across all SNPs. The best parameter combination from the parameter grid was defined as the one where the negative log-likelihood was minimized.

#### Interpolation to find the truly best parameters

Because only a relatively coarse grid of parameter combinations could be tested because of the computational demands of the analysis, we interpolated the likelihood surface to find the best parameter combination, including combinations not included in the grid. We used the Mathematica function Interpolation, which uses cubic spline. *f* (the number of generations per year) was fixed at 2, as this value produced the highest likelihood, while the parameter overall seemed to have little effect (figs. S8 to S10 and table S5).

#### Simulations to determine the “expected range” of allele frequency change without selection for each locus

We ran simulations to test whether the allele frequency changes observed in the control SNPs and the spatial outlier SNPs are consistent with a neutral model, given our model uncertainty and sampling procedure. For each SNP, we ran 1000 replicate simulations, always using the allele frequency observed in Crab in 1992 and the allele frequency observed in the Wave reference for the respective SNP as input. For each simulation, the combination of demographic parameters was randomly sampled from the likelihood surface (as described in the previous section). Each simulation started with the allele frequencies observed in the Crab sample in 1992 and included population growth (based on *r* and *K*) and random binomial sampling under gene flow and drift (based on *M*, *p_W_*, and the population size based on the growth parameters) in each generation. The simulations were stopped at the last sampling time point (2021).

We then determined the expected range of allele frequency change (i.e., frequency in 2021 – frequency in 1992) for each SNP as the range from the 2.5 to the 97.5% quantile of the distribution of simulated allele frequency changes in 2021. This expected range includes the effects of genetic drift, sampling, and the uncertainty of our model inference.

If the control SNP set contained no selected SNPs and the inference worked perfectly, ~5% of the control SNPs would fall outside this expected range and about half of the control SNPs would fall above the median of their respective expected range. In contrast, the spatial outlier SNPs were predicted to fall outside the expected range more frequently and more than half of them were predicted to fall above the median of their respective expected range, reflecting selection.

We ran the same analysis for the inversions, treating each inversion as a single locus and asking whether the arrangement frequency change exceeds the expected range. For complex inversions with three arrangements (LGC6.1/2 and LGC14.1/2), we included only the arrangement that was most common in the Wave population.

### Change in time versus space in collinear loci

To compare differentiation in space and in time, we calculated the *F*_ST_ between Crab and Wave populations based on previous studies (spatial *F*_ST_) and the *F*_ST_ between the skerry in 1992 and 2021 (temporal *F*_ST_) for the spatial outliers in the reduced-LD spatial outlier dataset. We predicted that SNPs with a more pronounced spatial differentiation also change more dramatically over time.

For the spatial outliers obtained from Morales *et al.* (*34*) (*n* = 37 SNPs), the spatial Crab-Wave *F*_ST_ was calculated by averaging across the Crab-Wave *F*_ST_ estimates for the locations Arsklövet, Saltö, Ramsö, and Jutholmen in that study. These locations are all in the same archipelago as the skerry system. For the spatial outliers obtained from Westram *et al.* (*33*) [*n* = 5 SNPs; does not include all spatial outliers from (*33*) because of missing data for some SNPs], the Crab-Wave *F*_ST_ was calculated by averaging across the Crab-Wave *F*_ST_ estimates for all seven hybrid zones included in Westram *et al.* (*33*) and Westram *et al.* (*23*), which are also located in the same archipelago. We then correlated spatial and temporal differentiation.

*F*_ST_ as typically used is not directional, i.e., the value does not indicate which of the two alleles is more common in which population. As we wanted to test whether the allele more common in Wave in space is the allele increasing on the skerry over time, we used signed *F*_ST_ values, setting the temporal *F*_ST_ to its negative if the allele more common in Wave in space decreased over time.

### Inversion frequency estimation

We determined the inversion genotype of each individual through a clustering analysis of individuals based on PCA of SNPs in each inversion separately. We performed a PCA on the filtered dataset, one inversion at a time including all samples, using the R package FactoMineR version 2.8 with default parameters (*66*). Because the first two principal components (PC) explain the largest percentage of the variance, individuals that are homozygous for each arrangement (homokaryotype) will cluster on the extremes of the PC1 versus PC2 plot, while heterozygous individuals (heterokaryotype) will cluster in-between the extremes (fig. S11) (*31*).

To identify the most likely cluster (i.e., inversion karyotype) for each individual in a population (points on the PC1 versus PC2 scatter plot), we used the implementation of k-means clustering algorithm in the R package stats version 4.3.0 with parameters nstart = 20, and centers = 3 for simple inversions and 6 for complex inversions. We validated the clustering patterns visually. Considering an inversion as a multi-allele locus, we estimated the frequency of each arrangement (not to be confounded with karyotype) within a population (e.g., Skerry 1992) by simply counting the number of alleles within the clusters where the arrangement occurs divided by the total number of alleles in the population. Last, we identified the arrangement that is more frequent in the Wave than in the Crab ecotype (based on merged samples from 2018 and 2021), which henceforth we will refer to as the Wave arrangement. The arrangement frequencies are summarized in table S6.

### Simulation of inversion trajectories within the expected range of frequencies without selection

To identify the arrangements that most likely increased in frequency due to selection, we generated a neutral range expectation of arrangement frequency trajectories. Using the parameters that best describe the demographic history of the skerry (table S7 and Supplementary text) and the initial arrangement frequencies in 1992, we simulated 1000 replicates of neutral trajectories of each inversion under drift and migration. In every generation *T* (of 58 until 2021, the last sampling time point), we randomly sampled *N_T_* alleles (population growth based on *r* and *K* from the best model) from a binomial distribution with a probability equal to the frequency of the Wave arrangement in *T*−1 plus the proportional contribution of migrants *M*. We determined the expected range for each inversion as the range from the 2.5 to the 97.5% quantile of the distribution of simulated arrangement frequencies in 2021. Thus, arrangements whose frequency in 2021 exceed the expected range are likely under selection. Figure S12 shows the observed and simulated trajectories of simple and complex inversions.

### Change in time versus space in inversions

To compare differentiation for inversions in space and in time, we estimated *F*_ST_ between Crab and Wave populations based on the arrangement frequencies for each inversion separately using genotypic data from the seven hybrid zones in four Swedish islands analyzed in Westram *et al.* (*33*) and Westram *et al.* (*23*) mentioned above. To estimate the arrangement frequencies, 30 individuals from both ends (Wave and Crab) of each transect were used in these estimates except for the Crab population from island CZB and CZD, where fewer individuals were available. As for the estimates of the inversion frequencies in the skerry, individuals with more than 20% of missing genotypes and SNPs with more than 20% missing data within each population were excluded. Individuals were genotyped for each inversion as in previous studies (*31*). Briefly, a PCA based on the diagnostic SNPs for each inversion was implemented using the R package PCADAPT (*67*) for individuals from each island, separately. The presence of an inversion was confirmed by the observation of three groups based on PC1 or six groups based on both PC1 and PC2 for complex inversions. The R function Kmeans was then used to identify the most likely individual genotype for each inversion using default settings for *K* = 3 and *K* = 6 clusters for simple and complex inversions, respectively. The frequency of each arrangement for each population was then obtained as described for the Skerry above. As for SNPs, *F*_ST_ for each inversion was calculated by averaging across *F*_ST_ between Crab and Wave estimates for all hybrid zones and subsequently used in the correlation with temporal differentiation.

### Use of AI-assisted technologies

AI-powered tools ChatGPT (version 3.5) and Bard (version 1.37) were only used to identify and correct grammatical errors and improve the clarity of the writing in some cases. The full prompts, e.g., “Please correct wordiness and grammar of this statement,” were used for editing the statement.

## Supplementary Materials

### The PDF file includes:

Supplementary Text Figs. S1 to S19 Tables S1, and S3 to S13 Legend for table S2 Legend for code S1 PDF version of Mathematica Workbook: *Skerry interpolation 9.23 v2.nb*

### Other Supplementary Material for this manuscript includes the following:

Table S2

Code S1
