# Supplementary material for: Predicting rapid adaptation in time from adaptation in space: A 30-year field experiment in marine snails
Source: Sci Adv. 2024 Oct 11;10(41):eadp2102. doi: 10.1126/sciadv.adp2102 (PMC11808926; doi:10.1126/sciadv.adp2102)
Supplement: Supplementary file 1 — Supplementary Text Figs. S1 to S19 Tables S1, and S3 to S13 Legend for table S2 Legend for code S1 PDF version of Mathematica Workbook: Skerry interpolation 9.23 v2.nb [file sciadv.adp2102_sm.pdf]

Science Advances
AAAS

Supplementary Materials for

Predicting rapid adaptation in time from adaptation in space: A 30-year field experiment in marine snails

Diego Garcia Castillo *et al.*

Corresponding author: Diego Garcia Castillo, diegofernando.garciacastillo@ist.ac.at;
Anja M. Westram, anja.m.westram@nord.no

Sci. Adv. **10**, eadp2102 (2024)
DOI: 10.1126/sciadv.adp2102

The PDF file includes:

Supplementary Text
Figs. S1 to S19
Tables S1, and S3 to S13
Legend for table S2
Legend for code S1
PDF version of Mathematica Workbook: *Skerry interpolation 9.23 v2.nb*

Other Supplementary Material for this manuscript includes the following:

Table S2
Code S1

Supplementary Text

Results of selection estimates based on phenotypes

Using V_g-*crab*, estimates of the strength of stabilising selection (V_s/V_p) ranged from 1.65 to 7.84 on the assumption of one generation per year and from 3.80 to 25.3 on the assumption of two generations per year, excluding the aperture position trait (*r0*) for which all of the change on the skerry could be accounted for by plasticity (Table S2). Weaker selection was inferred using the larger estimate of genetic variance (V_g-*site*). The strongest selection was on width growth (*gw*), for which the skerry phenotype was already close to the Wave reference phenotype in 2002. Other traits were approaching the reference phenotype in the final two samples (2018 and 2021). The estimated strengths of stabilising selection are in the range observed in surveys of phenotypic selection in nature, where V_s/V_p is typically ~5 (*45*, *47*). However, this translates into strong selection on the Crab phenotype in the skerry environment because of its distance from the optimum, with some estimated fitness reductions >90% under the assumption of one generation per year and using the V_g estimate for the Crab populations (Table S2).

Results of the demographic inference

Estimated demographic parameters

The most likely combination of parameters from the grid is shown in Table S7. The means and support limits for the different parameters based on interpolation are shown in Table S5. Note that the parameters referring to counts of individuals reflect haploid individuals; the diploid value would be half the estimates shown here.

Expected range

For both control and spatial outlier SNPs, the proportions of SNPs inside and outside the expected range, and above and below the median value from 1,000 replicate simulations are shown in Table S8 and illustrated in Figure S13. For the control SNPs, we found that 10% of SNPs were outside the expected range, i.e. slightly more than the 5% expected if the model fitted perfectly and the SNPs were only affected by gene flow and drift. The discrepancy is unsurprising given that some SNPs may be affected by linked selection and that it is unlikely that our simple model perfectly captures the history of the skerry. The pattern is more or less symmetrical (almost as much change away from Wave as towards Wave), in line with most SNPs evolving neutrally.

For spatial outliers, we found a similar proportion outside the expected range (9%); however, there was a clear asymmetry, with almost all SNPs outside the expected range showing more change towards the Wave allele frequency than expected, and with 67% of all SNPs (62% of the *reduced-LD spatial outliers*) showing more change towards Wave than the median change under neutrality. These results indicate that at least a large subset of the spatial outlier SNPs is affected by selection and this pattern is not the result of genomic clustering of spatial outliers in LD. However, it is difficult to say which exact SNPs are affected by selection, as the extent of drift and / or the model uncertainty are too high for most SNPs to show statistically significant changes (i.e. be outside the expected range).

For the inversions, we found that all 13 inversions included in the analysis showed an arrangement frequency change above the median of the respective expected range and towards Wave frequency, with 4 (31%) showing a change outside the expected range. These results strongly indicate that multiple inversions are affected by selection on the skerry.

Mathematical approximation to the origin of the adapted alleles

What is the relative contribution of standing variation vs gene flow? Suppose that alleles flow in by migration at a rate $m[t]$, which may vary through time; the source population has allele frequency p^*. We distinguish alleles that were originally present as standing variation, at frequency p_0, from those that immigrate later, denoting their frequency p_m, p_{sv} respectively. Selection s favours both classes of allele. Approximating to continuous time:

$$\partial_t p_m = m[t]\,(p^* - p_m) + s\,p_m(1 - p_m - p_{sv}), \qquad p_m[0] = 0$$

$$\partial_t p_{sv} = m[t]\,(-p_{sv}) + s\,p_{sv}(1 - p_m - p_{sv}), \qquad p_{sv}[0] = p_0$$

We can find a simple approximation to their relative contribution by considering their growth from low frequency (p_m, p_{sv} <, 1), and assuming that p_0, m/s are small. Then, once $spq >> m$, the contribution from migration becomes negligible, relative contributions of p_0 vs m remain in the same ratio. Therefore, we can look at the initial stage when $p << 1$:

$$\partial_t p_m = m[t]\,p^* + s\,p_m$$

$$\partial_t p_{sv} = s\,p_{sv}$$

$$p_m[t] = \left(\int_0^t m[\tau]\;p^*\,e^{-s\tau}\,d\tau\right)e^{st}$$

Thus, we see that the relative contributions would be in the ratio mp^*/s to p_0 for constant m. If we had a constant number of immigrants, M, and a population growing as $N_0 e^{rt}$, then

$m = (Mp^*/N_0)\,e^{rt}$, and we see that the ratio of contributions is $\frac{m_0 p^*}{r+s} = \frac{(M/N_0)p^*}{r+s}$ vs p_0. This prediction is not affected (on average) by random drift, because drift does not change the expected allele frequency in the initial growth phase

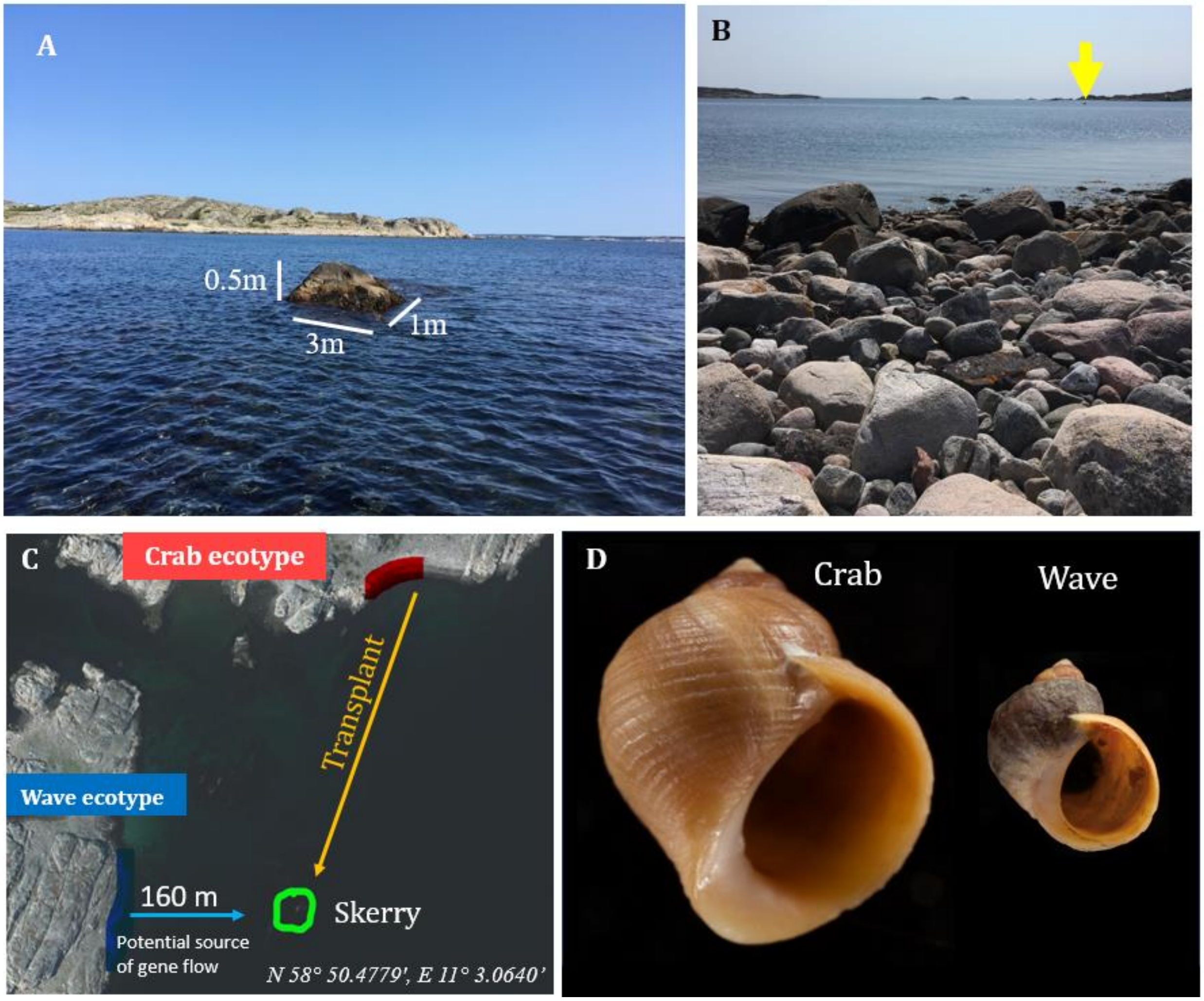

Fig. S1. The skerry area. (A) View and dimensions of the skerry, the target of the transplanted snails from a Crab ecotype. **(B)** A view of the skerry (yellow arrow) taken from the shore of the donor Crab ecotype. **(C)** A satellite view of the skerry, the donor Crab population, and the neighbouring Wave population. **(D)** Two sample shells from the Crab ecotype and the Wave ecotype.

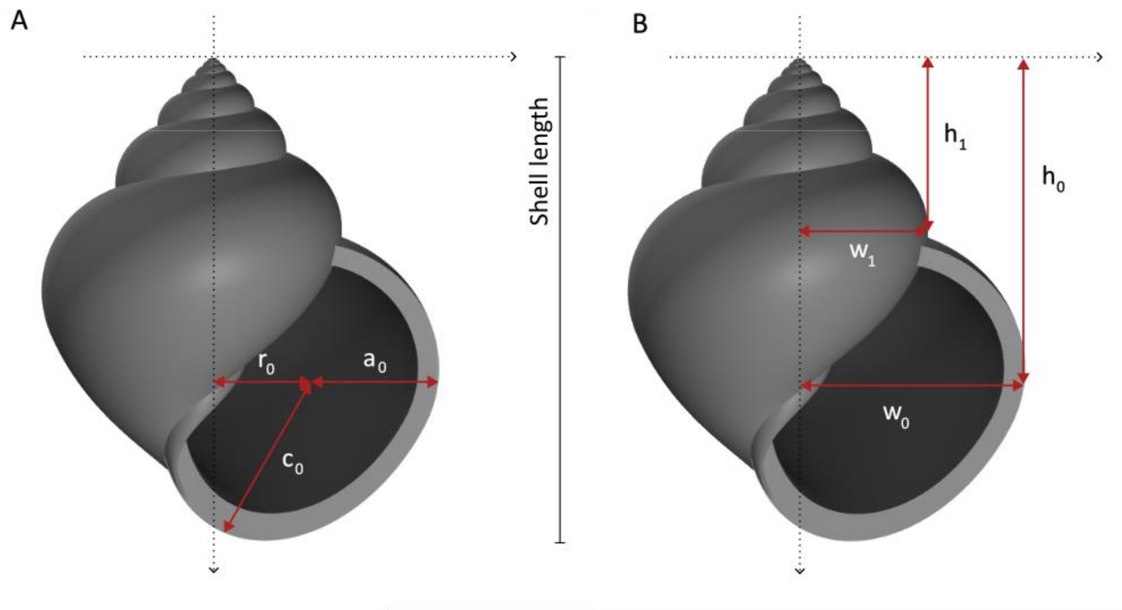

Fig. S2: Description of the shape parameters used in the analysis. All measurements are relative the total shell length, to remove the size component.
a_0 : Size of the (circular, upper part of the) aperture.
r_0 : Radial position of the aperture reference point (centre of the circular part).
h_0 : Vertical position of the aperture reference point
c : Shape of the aperture, measuring elliptical eccentricity of the lower part of the aperture ($\boldsymbol{c = \frac{c_0}{a_0}}$)
g_w: Width growth between consecutive whorls ($\boldsymbol{g_w = \frac{1}{2\pi} ln\left(\frac{w_0}{w_1}\right)}$).
g_h: Height growth between consecutive whorls ($\boldsymbol{g_h = \frac{1}{2\pi} ln\left(\frac{h_0}{h_1}\right)}$).

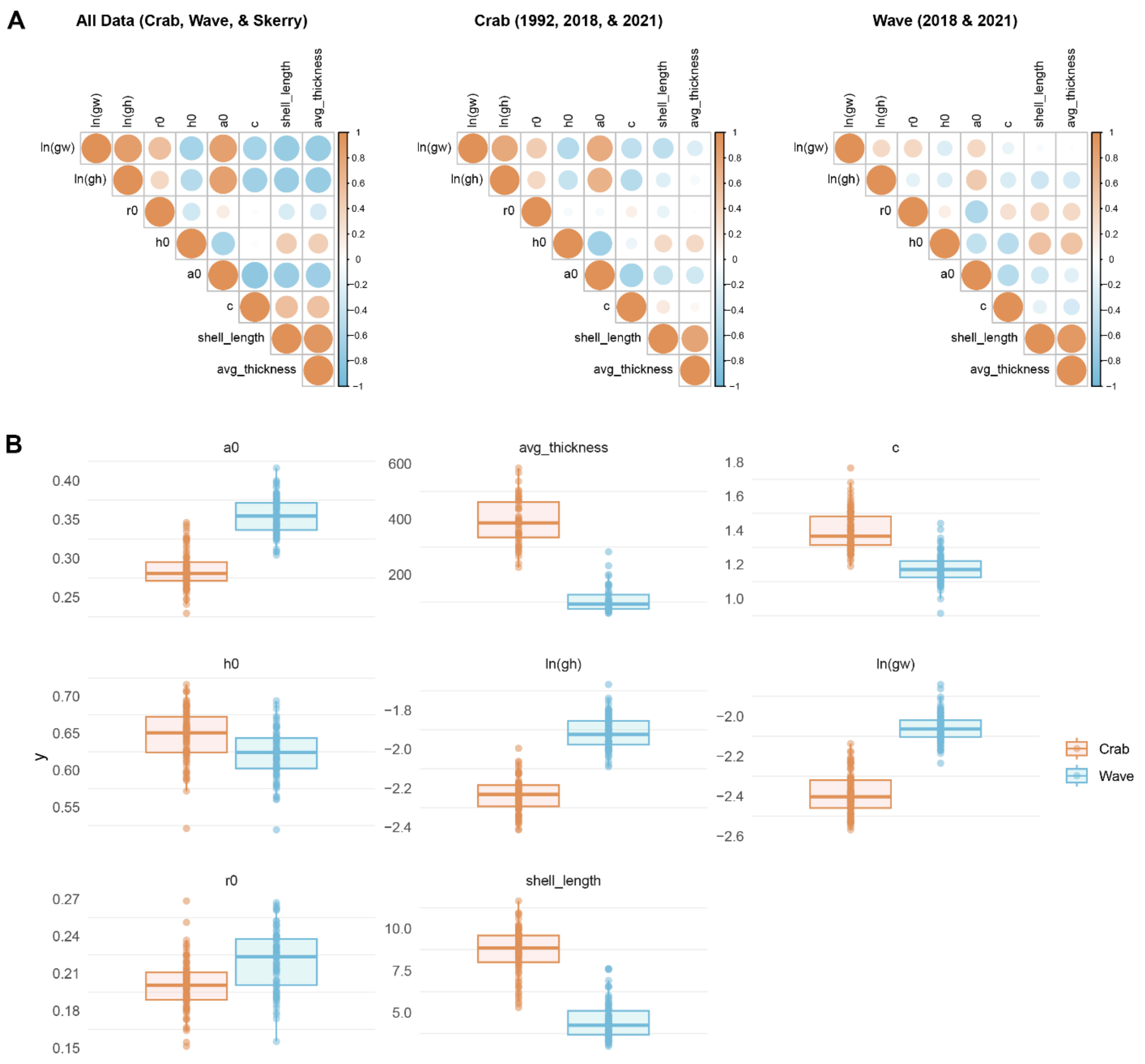

Fig. S3. Comparisons among quantitative traits. (A) Correlation plots of different pairs of quantitative traits in all skerry samples, the Crab ecotype, and the neighbouring Wave ecotype. The colour scale and the size of the circle are proportional to the correlation coefficient between each pair of traits. **(B)** Boxplots of pairs of diagnostic traits in the Crab ecotype and neighbouring Wave ecotype.

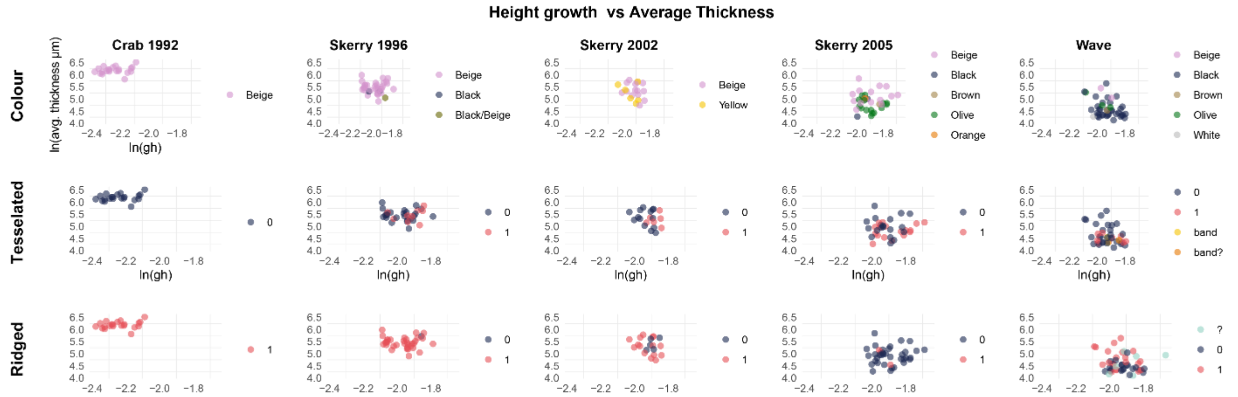
Height growth vs Average Thickness
Crab 1992
Skerry 1996
Skerry 2002
Skerry 2005
Wave
Colour
Tesselated
Ridged
ln(avg. thickness μm)
ln(gh)
Beige
Black
Black/Beige
Yellow
Brown
Olive
Orange
White
0
1
band
band?
?

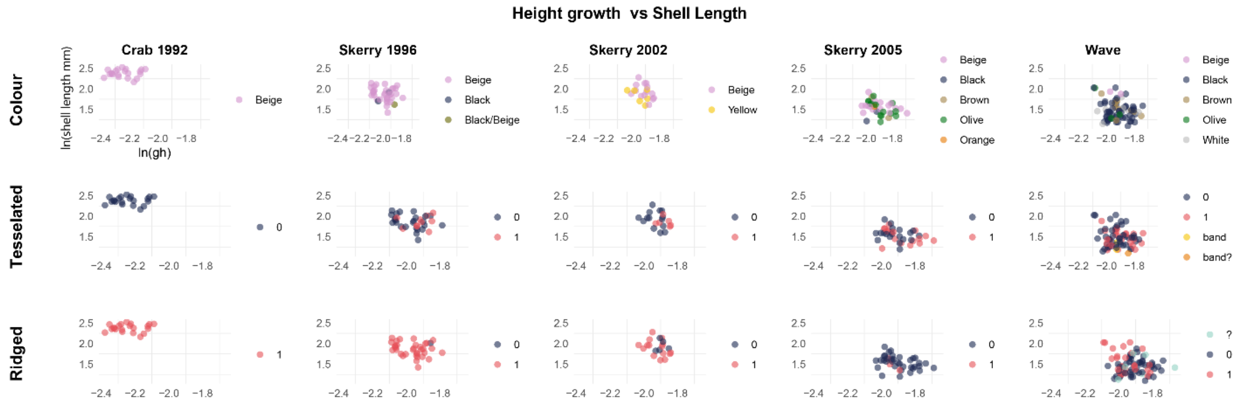
Height growth vs Shell Length
Crab 1992
Skerry 1996
Skerry 2002
Skerry 2005
Wave
Colour
Tesselated
Ridged
ln(shell length mm)
ln(gh)
Beige
Black
Black/Beige
Yellow
Brown
Olive
Orange
White
0
1
band
band?
?

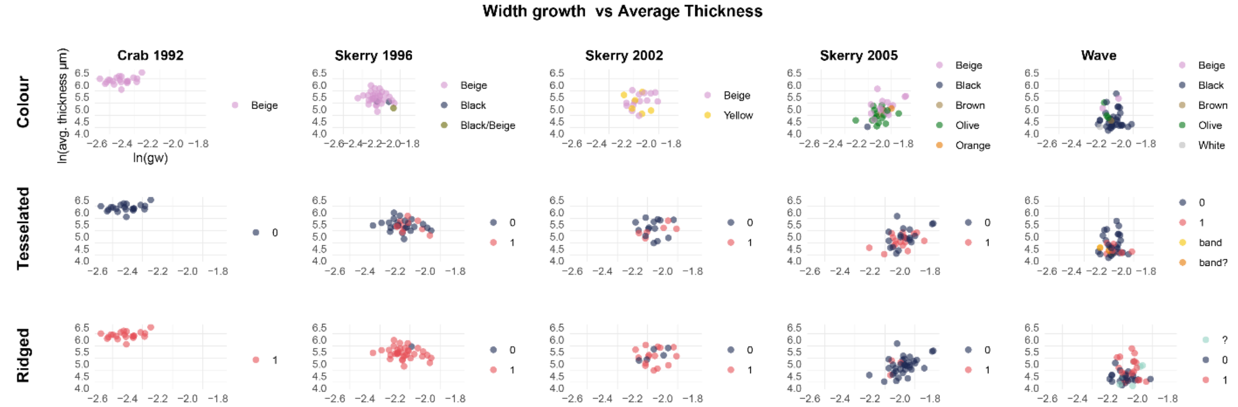
Width growth vs Average Thickness
Crab 1992
Skerry 1996
Skerry 2002
Skerry 2005
Wave
Colour
Tesselated
Ridged
ln(avg. thickness μm)
ln(gw)
Beige
Black
Black/Beige
Yellow
Brown
Olive
Orange
White
0
1
band
band?
?

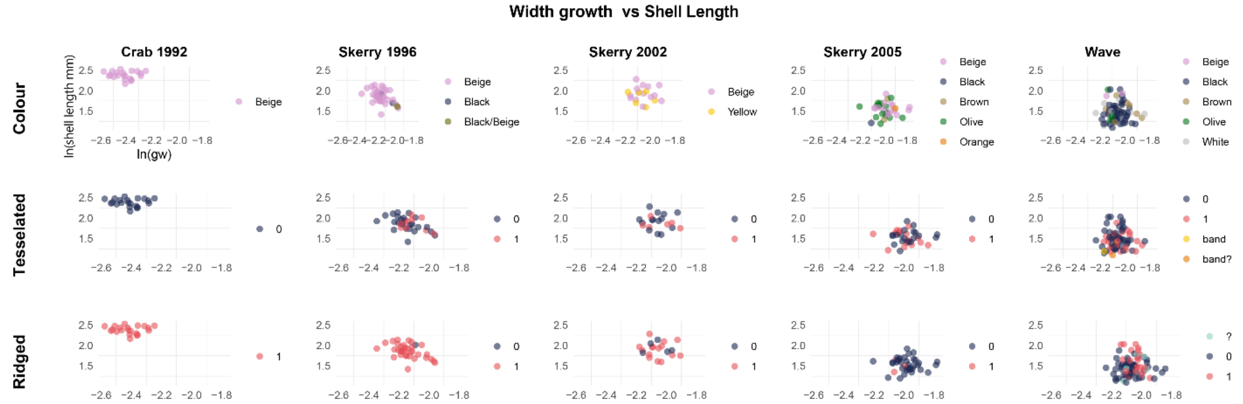
Width growth vs Shell Length
Crab 1992
Skerry 1996
Skerry 2002
Skerry 2005
Wave
Colour
Tesselated
Ridged
ln(shell length mm)
ln(gw)
Beige
Black
Black/Beige
Yellow
Brown
Olive
Orange
White
0
1
band
band?
?

Fig. S4. Scatter plot of pairs of uncorrelated quantitative traits for the first few years after the introduction. Rows are three groups of qualitative traits. Samples are coloured by the different categories of a qualitative trait. All four quantitative traits (average thickness, average shell length, height growth, and width growth) were transformed to natural logarithm (ln).

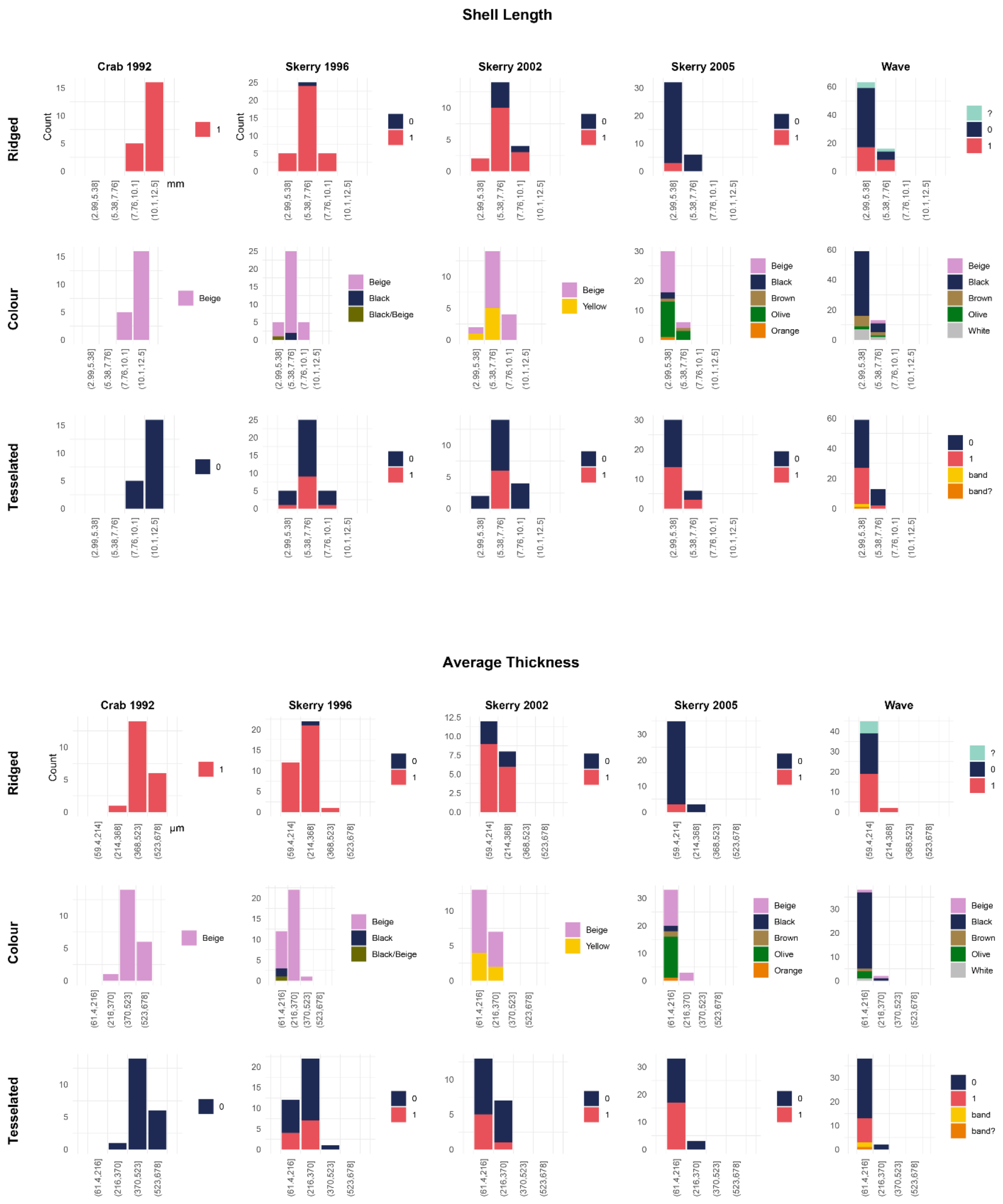

Fig. S5. Bar plots of four diagnostic traits (shell length, average thickness, width growth, and height growth) in the first few years after the introduction. Rows are three groups of qualitative traits. Bars were plotted in four bins, and coloured by the counts in different categories of a qualitative trait within each bin. Width growth and height growth are the log-transformed values *ln(gw)* and *ln(gh)*. This figure continues on the next page.

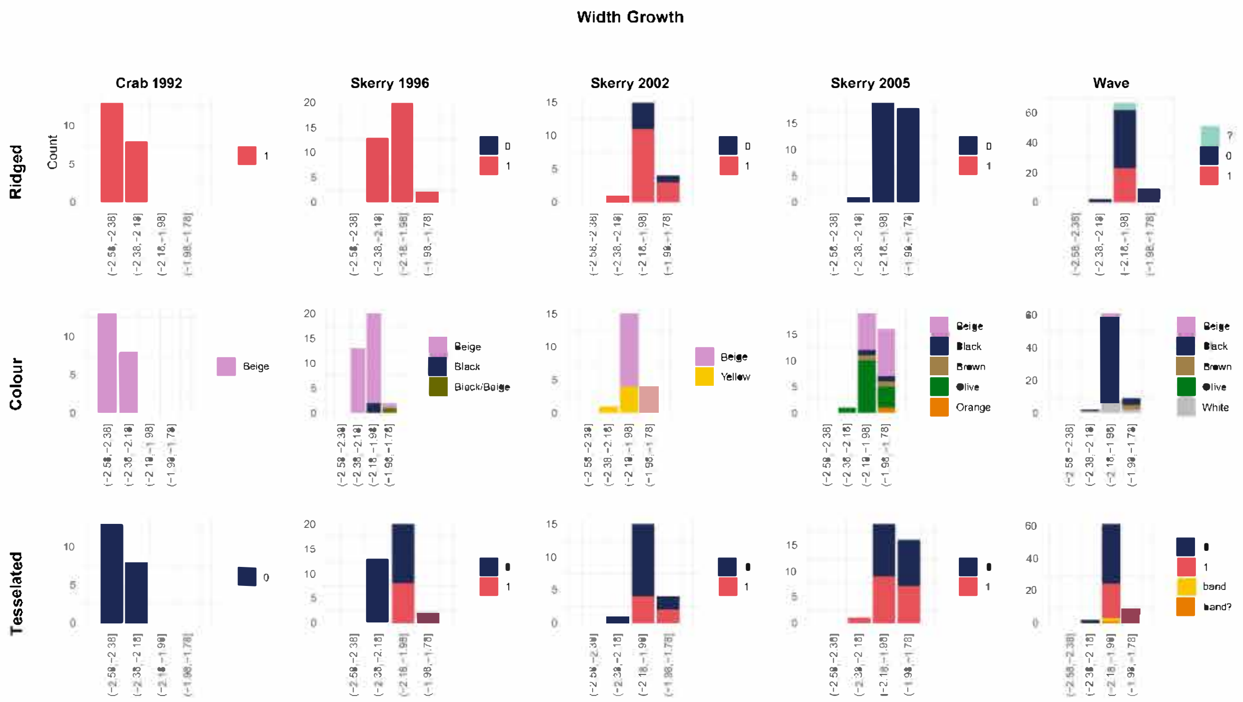
Width Growth
Crab 1992
Skerry 1996
Skerry 2002
Skerry 2005
Wave
Ridged
Colour
Tesselated
Count
Beige
Black
Brown
Olive
Orange
White
Yellow
Black/Beige
band
band?

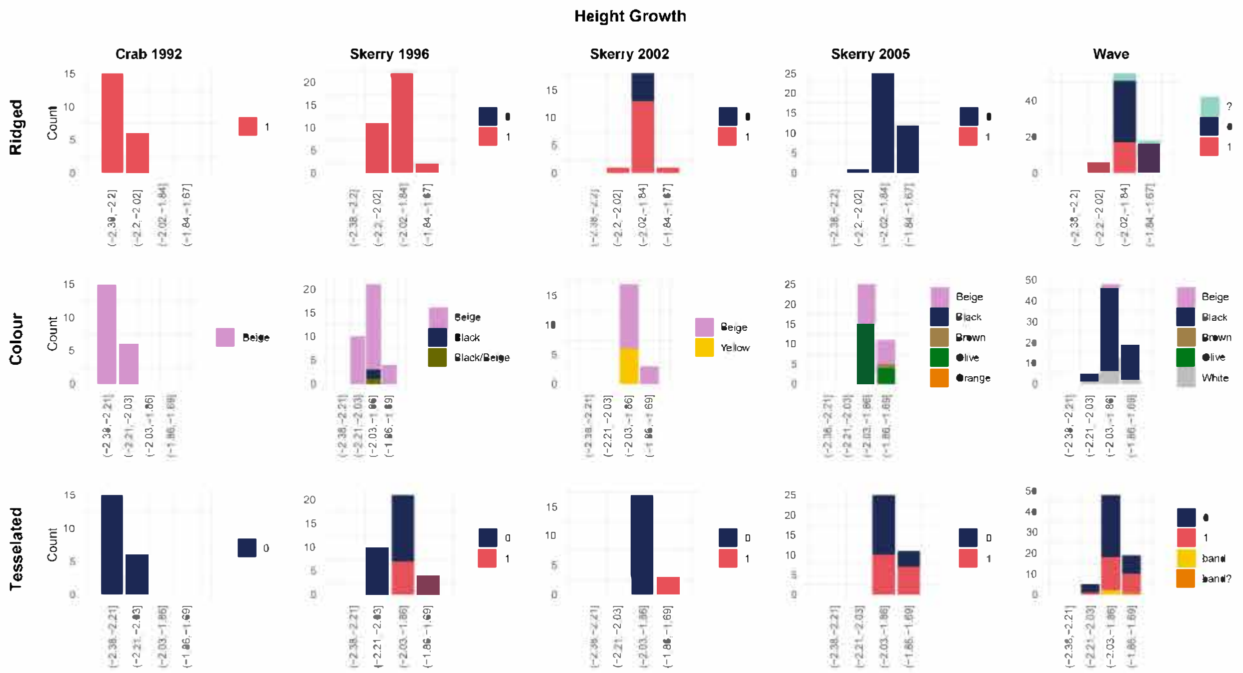
Height Growth
Crab 1992
Skerry 1996
Skerry 2002
Skerry 2005
Wave
Ridged
Colour
Tesselated
Count
Beige
Black
Brown
Olive
Orange
White
Yellow
Black/Beige
band
band?

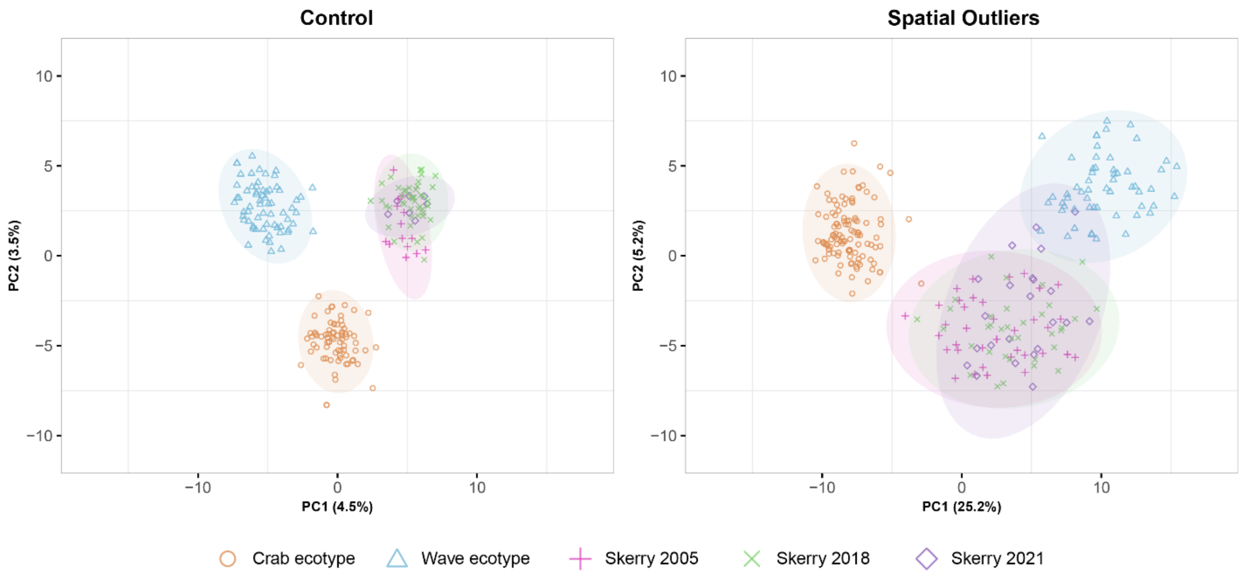

Fig. S6. PCA on collinear loci. Crab ecotype (donor Crab) includes the samples from 1992, 2018, and 2021. Wave ecotype includes the samples from 2018 and 2021. The clustering pattern of control loci suggests a strong effect of drift that separates genetically the skerry from the donor population (Crab) on both axes. On the contrary, for candidate outliers (*full spatial outlier dataset*), the skerry samples cluster closer to the Wave ecotype on PC1 (the axis that explains a larger proportion of the total variance) likely due to a strong effect of selection. This plot also shows that most change on the skerry happened early in the experiment, as there is little change from 2005 to 2021.

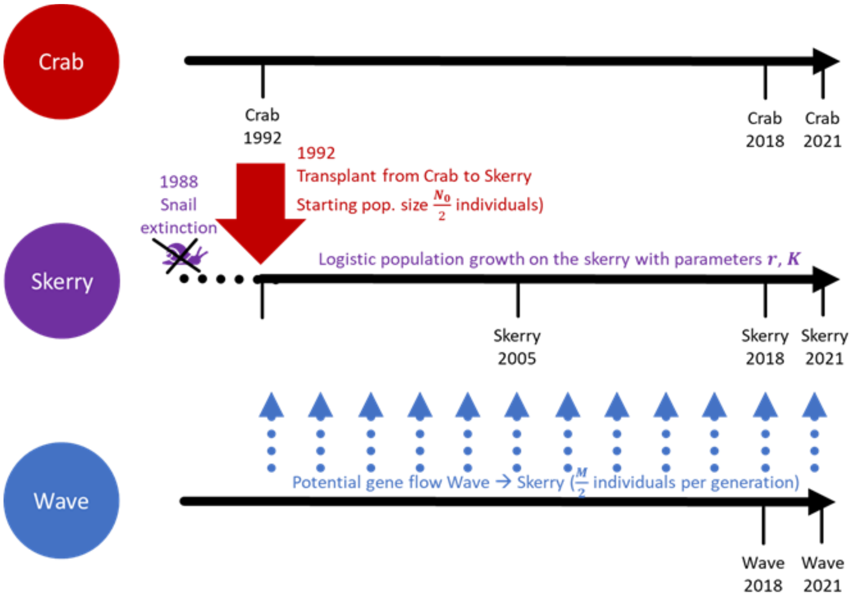

Fig. S7: Schematic depiction of the experiment, with parameters of the demographic model indicated in bold. The black arrows represent time; sampling times are indicated below. Events important for the experiment are highlighted in colour.

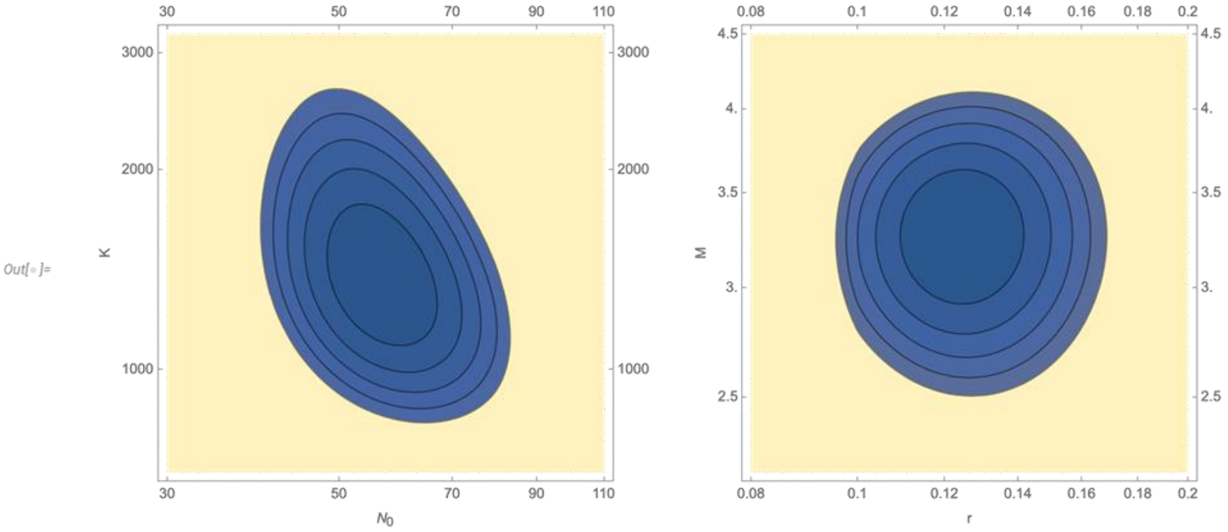

Fig. S8. Contours of log likelihood, with spacing 1, as a function of N_0, K (left) or r, M (right). In each plot, the other two parameters are fixed at their maximum-likelihood estimate (left: r=0.12, M=3.25; right: N_0=55.4, K=1371). For two degrees of freedom, a loss of log likelihood of 3 corresponds to $\chi_2^2 = 6$, or P=5% (see below). Thus, three contours down corresponds to a 95% confidence interval.

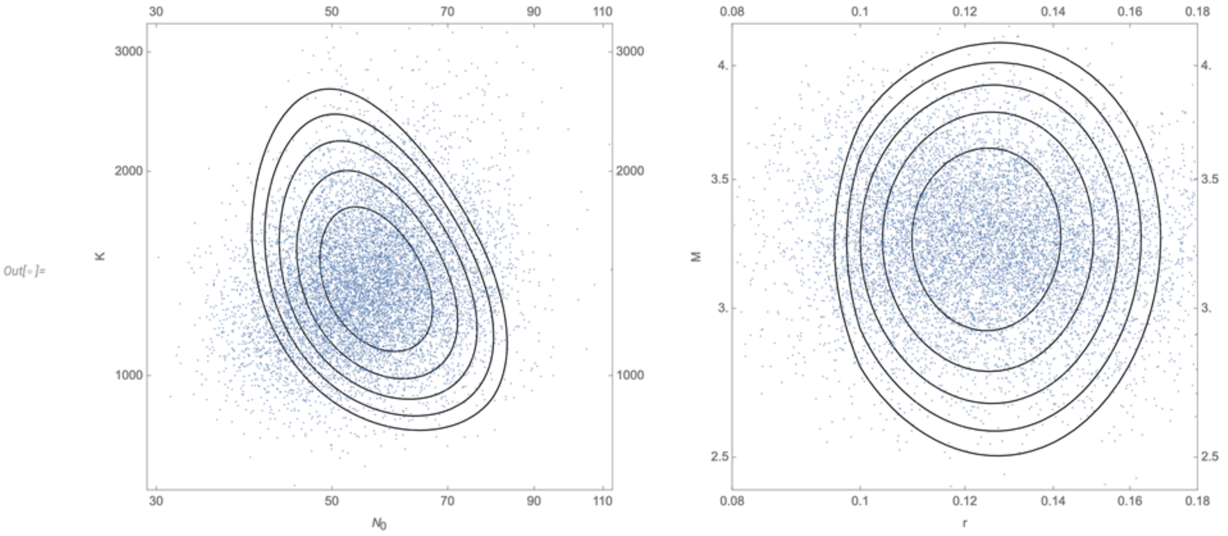

Fig. S9. **Posterior distribution (points) superimposed on contours of log likelihood (spacing 1).** Left: N_0 vs K. right: r vs. M. Contours on the left plot fix f=2, M=3.25, r=0.12; the right plot fixes N_0=55.4, K=1371. Note that these distributions are not quite the same: each posterior distribution averages over the posterior distribution of the other two parameters, whereas the contours show the log likelihood with the other two parameters fixed at their MLE.

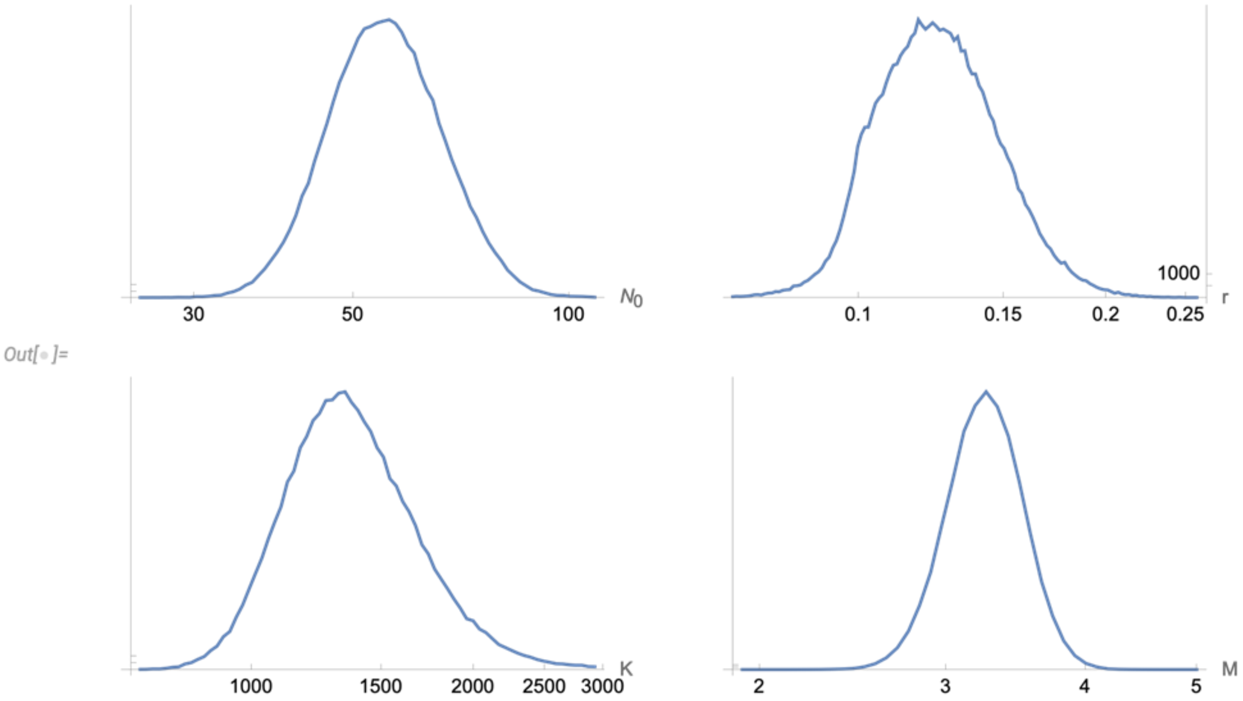

Fig. S10. Posterior distribution for the four parameters, based on 500,000 random draws, using the Metropolis algorithm.

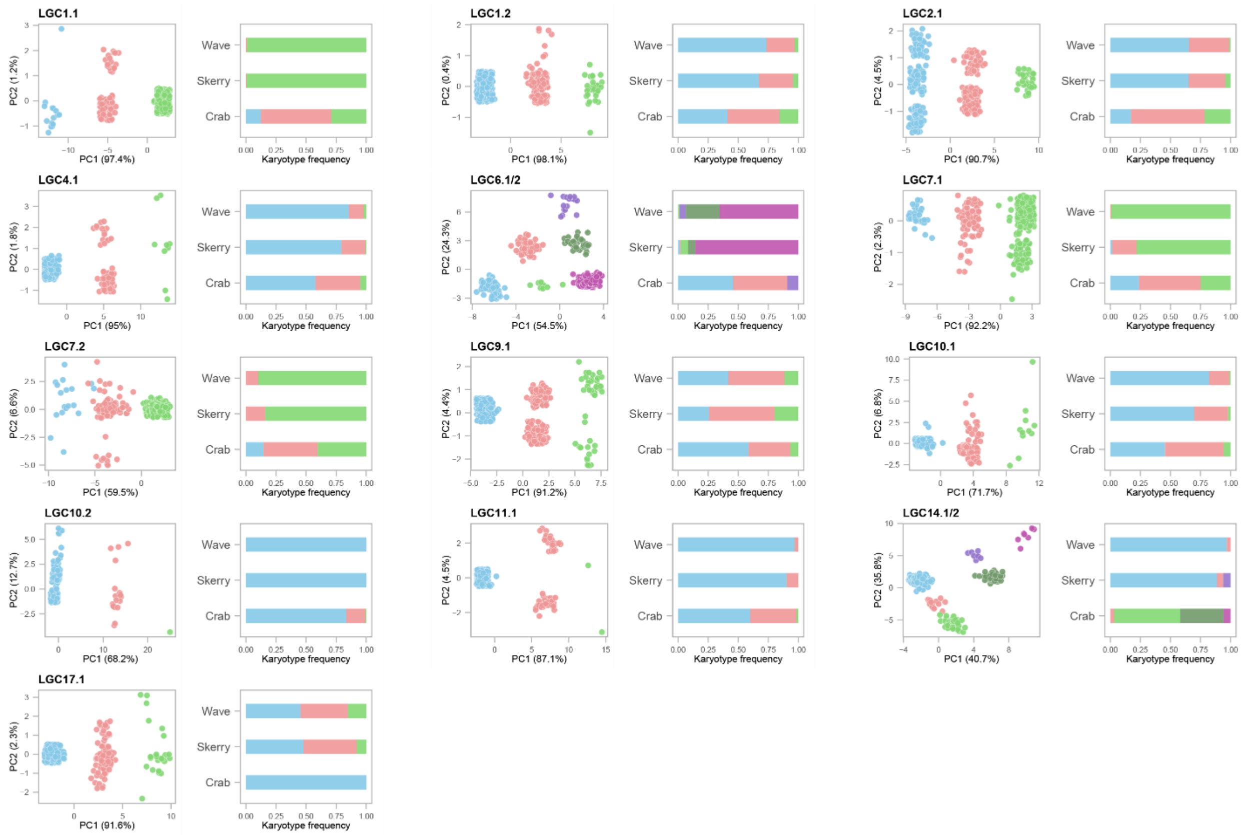

Fig. S11. Clustering pattern of PC1 vs PC2 for both simple and complex inversions. The bar plots next to each scatterplot show the frequencies of the different karyotypes for a particular inversion in the skerry, Crab (1992, 2018, and 2021) and Wave (2018 and 2021) ecotype populations. The points were coloured based on the most likely partitioning by a k-means algorithm. The colours in the bars correspond to the colours of the clusters in the scatterplot.

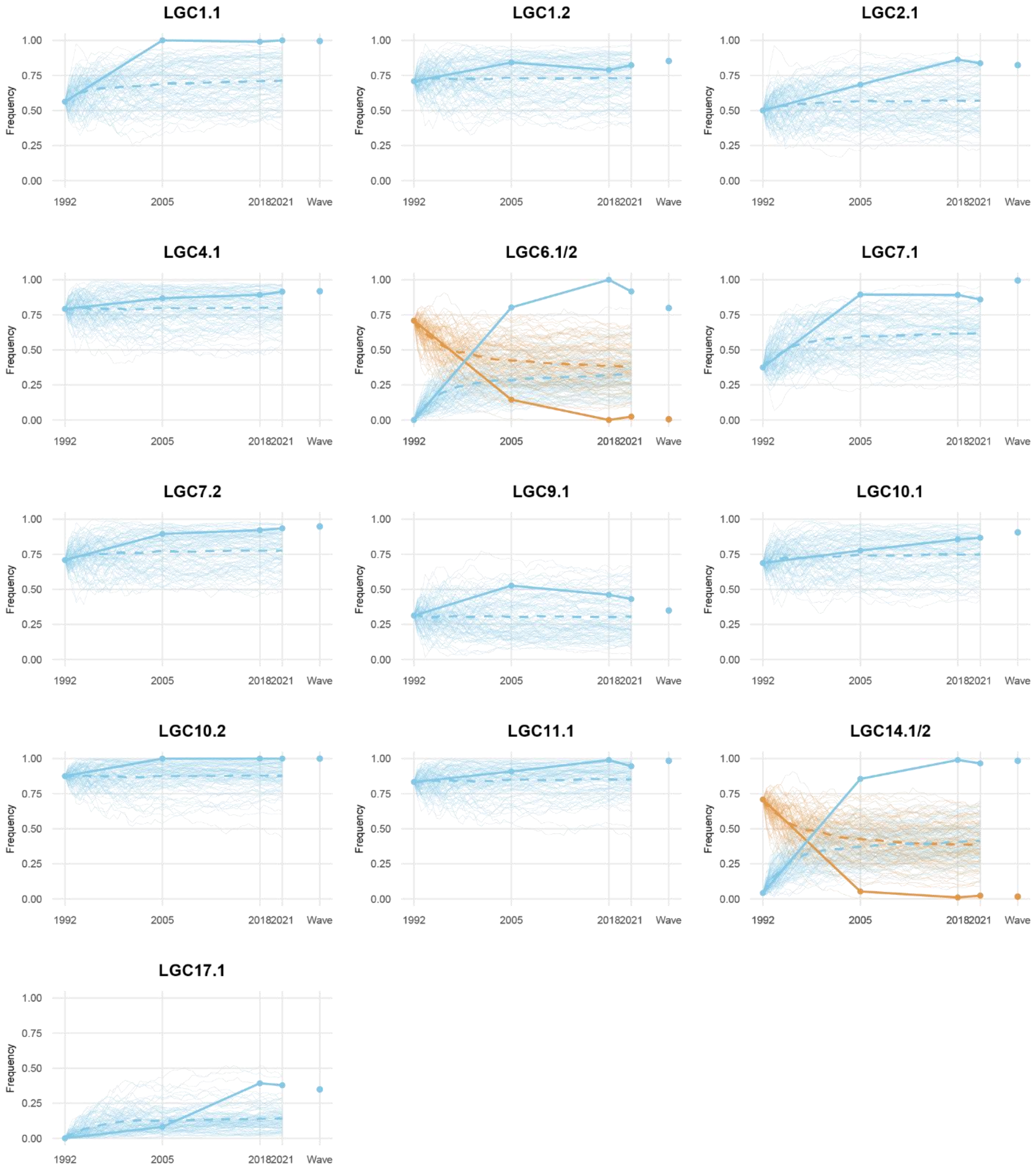

Fig. S12 Frequency trajectories of the Wave arrangement. In blue, trajectories of the Wave arrangement. In orange, trajectories of the Crab arrangement in complex inversions (two out of three arrangements). To facilitate visualization, the figures include 100 replicates of simulated trajectories for each arrangement (thin and pale lines). However, the neutral range was generated from 1,000 simulated trajectories for each arrangement.

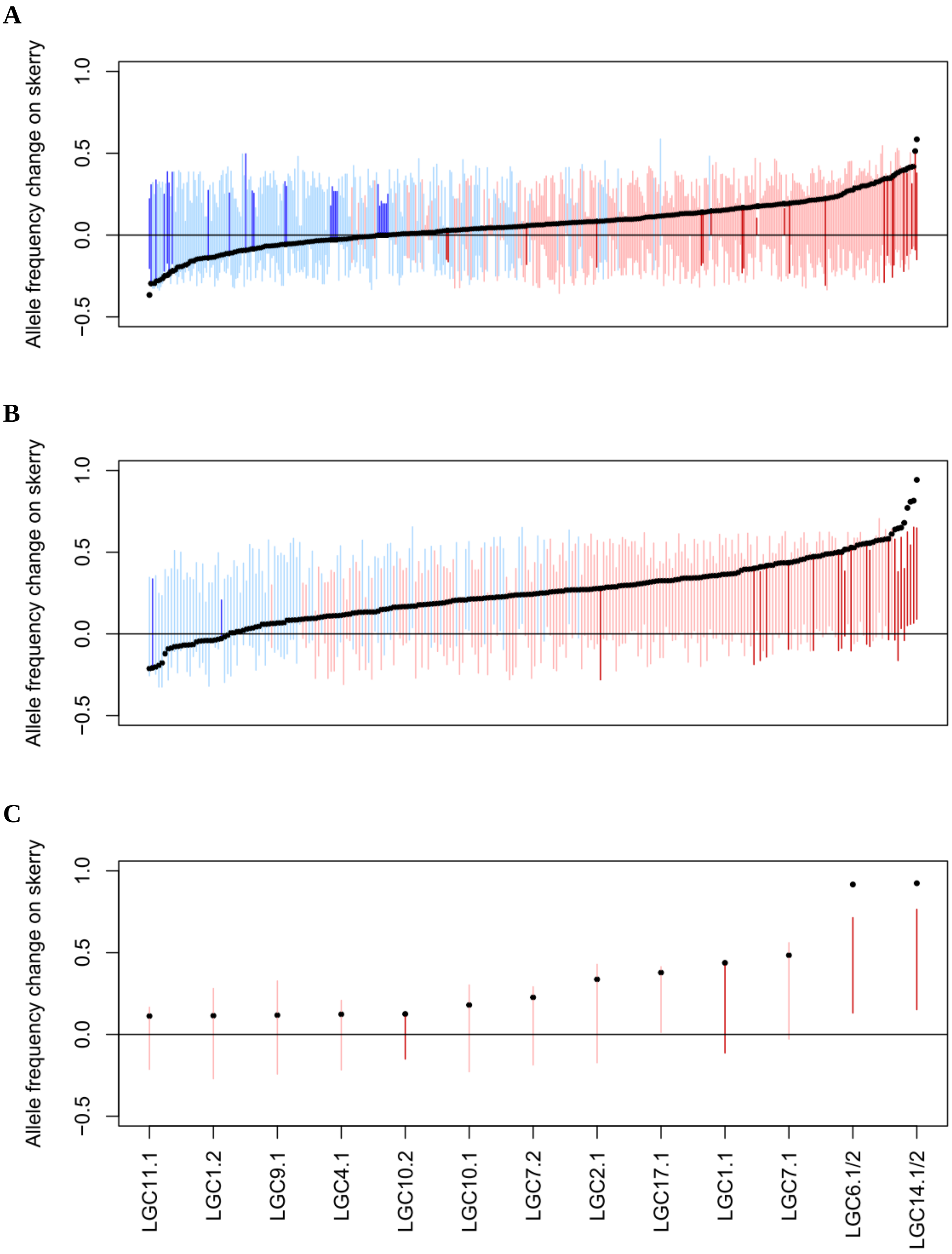

Fig. S13. Expected ranges (coloured bars) and observations (black circles) for the allele frequency change on the skerry (i.e. the allele frequency difference between the skerry population in 2021 and the Crab donor population in 1992). Positive allele frequency change indicates change towards the allele more common in Wave, while negative change indicates that

the frequency of the allele more common in Wave decreased. Loci are sorted along the x-axis by the extent of allele frequency change. A) control SNPs; B) spatial outlier SNPs (*full spatial outlier dataset*); C) inversions. Dark blue: observation outside expected range and below median; light blue: observation inside expected range and below median; light red: observation inside expected range and above median; dark red: observation outside expected range and above median. For the inversions, inversion IDs are indicated along the x-axis. For complex inversions with three arrangements, only the arrangement most common in Wave is shown.

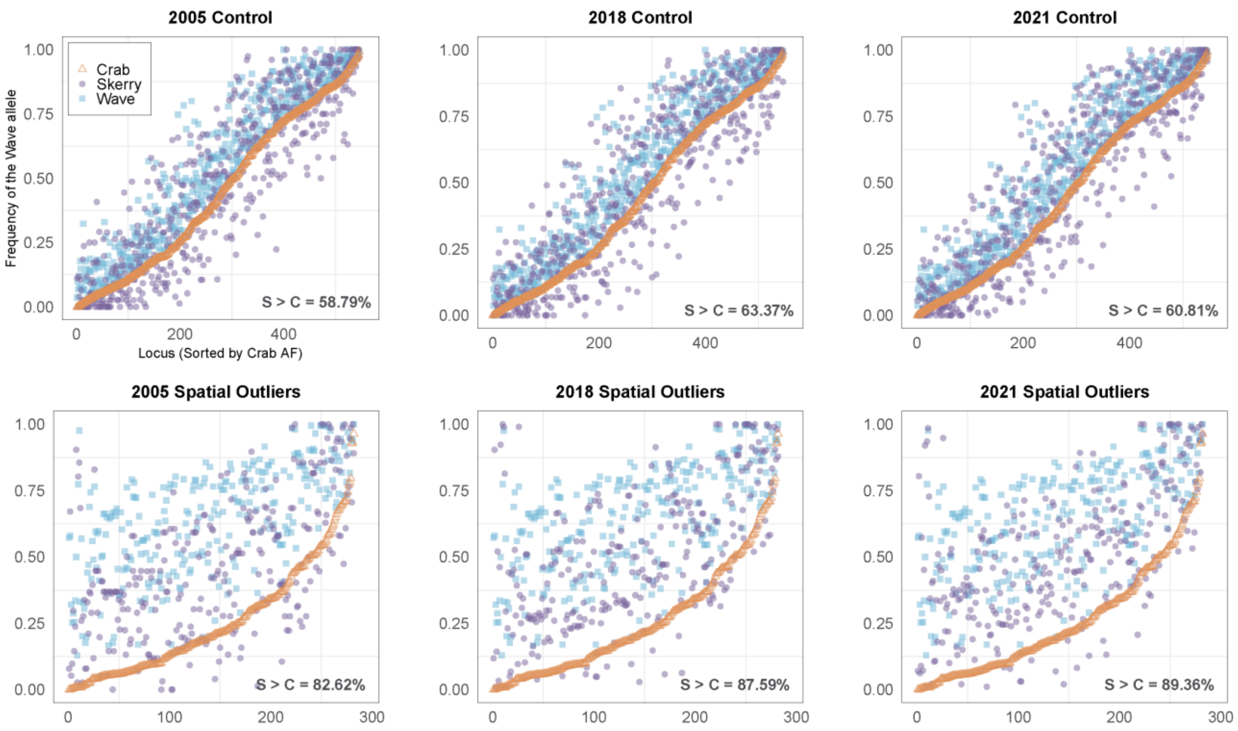

Fig. S14. Frequency of the Wave allele in the skerry and reference populations. "Wave" allele refers to the allele that is more common in the Wave (merged samples from 2018 and 2021) than in the Crab (merged samples from 1992, 2018, and 2021) ecotype populations. We sorted the loci in ascending order, according to the allele frequency (AF) in Crab. S > C is the percentage of SNPs in skerry with Wave allele frequency greater than the frequency in Crab. In all sampled years, the frequency of the Wave allele in skerry was more commonly observed above the frequency found in Crab (the orange middle pattern is the result of adjacent SNPs sorted by AF in crab). As expected due to their role in ecotype divergence, spatial outliers (bottom row) changed more frequently towards the Wave frequency compared to control loci (top row). The *full spatial outlier dataset* was used.

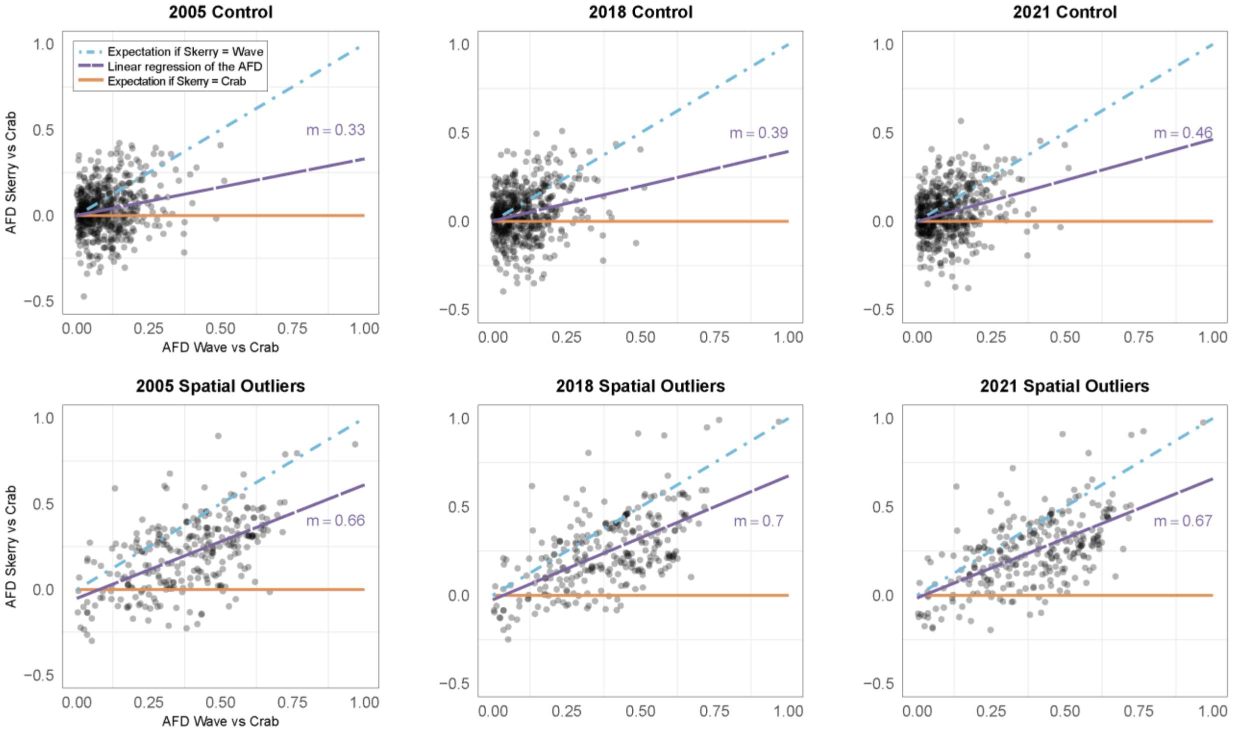

Fig. S15. Directional allele frequency changes in the skerry in three sampling years. Allele frequency difference (AFD) estimates are based on the frequency of the "Wave" allele (allele that is more common in Wave than in Crab). The allele frequencies of Wave included samples from 2018 and 2021. Likewise, the allele frequencies of Crab included samples from 1992, 2018, and 2021.The slope (m) of a linear regression (dashed line) indicates a directional allele frequency change towards Wave in spatial outliers (bottom row) but not in control loci (top row), as predicted. The dashed blue line represents the expectation if the allele frequencies (AFs) in the skerry population were identical to those in the Wave ecotype. The solid orange line represents the expectation if the AFs in the skerry population were identical to those in the Crab ecotype. The *full spatial outlier dataset* was used.

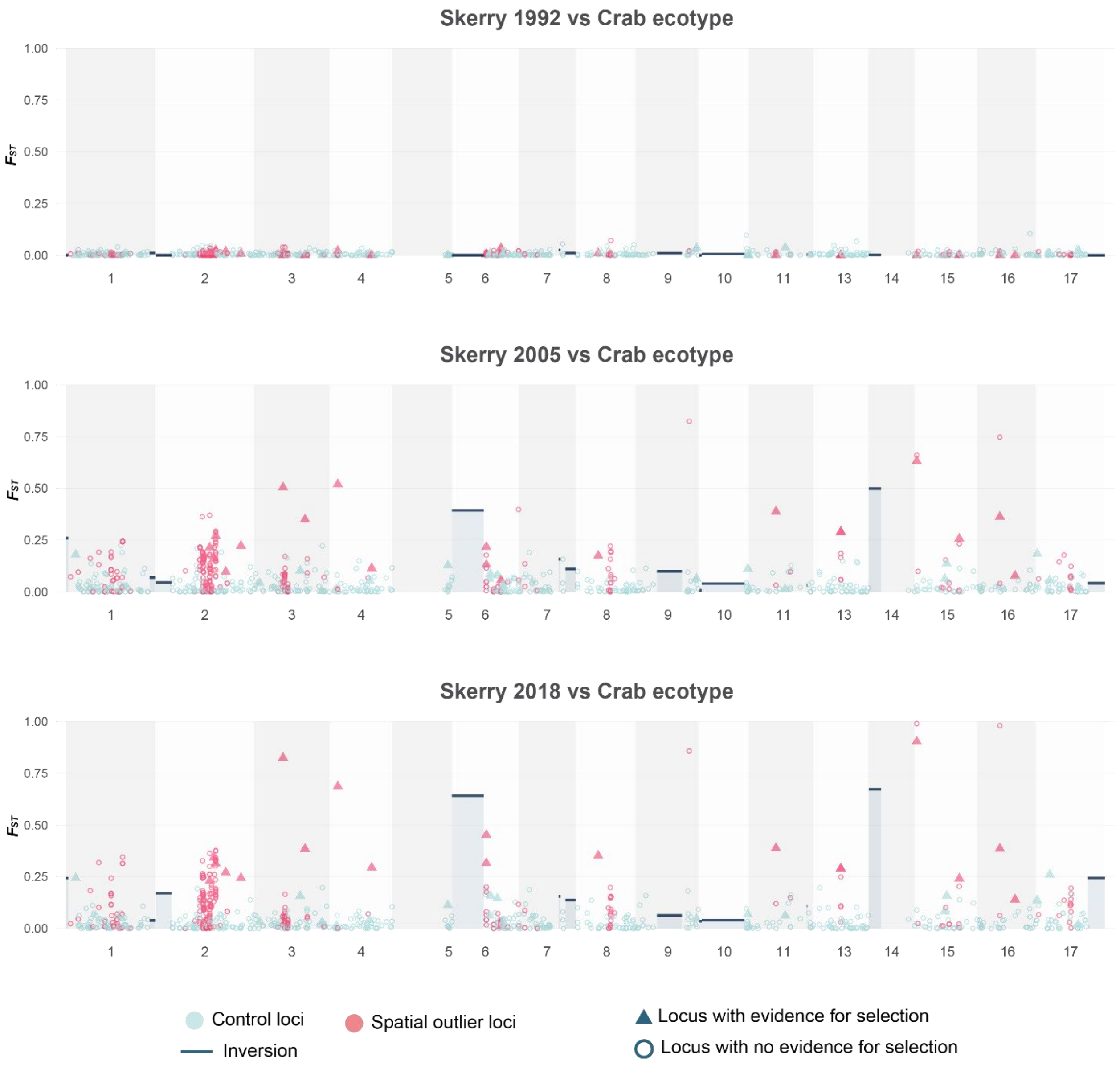

Fig. S16. Genome-wide F_{ST} in the skerry versus the combined samples of the Crab ecotype (2018+2021) in three different years. Circles and triangles represent individual SNPs in the collinear genome. Inversions are represented by rectangular blue-grey fields with black bars at the top indicating F_{ST} value. The *full spatial outlier dataset* was used. The results for the year 2021 are shown in the main text (Fig. 2D).

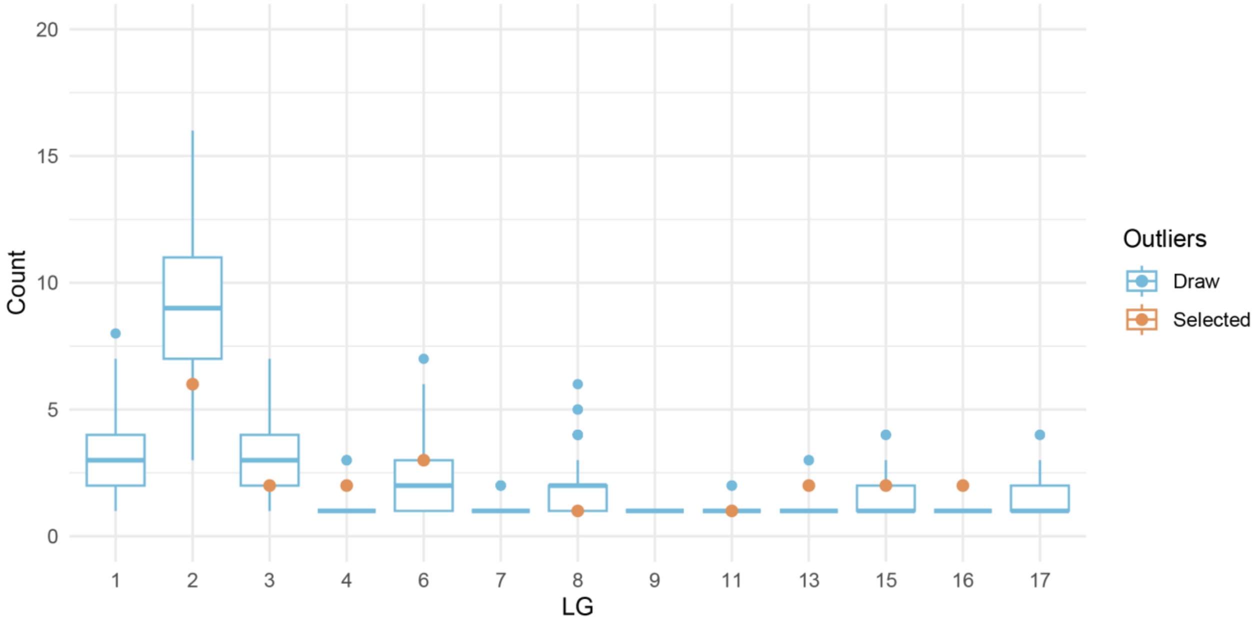

Fig. S17. Count of spatial outliers with evidence for selection (orange) with respect to the expectation based on SNP content in each chromosome (blue). We randomly sampled 21 spatial outliers (the number of spatial outliers with evidence for selection based on the expected range under neutrality) among all spatial outliers (*full spatial outlier dataset*) along the collinear genome 1,000 times. The random draws, which are the chance expectation, are plotted as blue boxes. Only linkage groups with more than one spatial outlier were included.

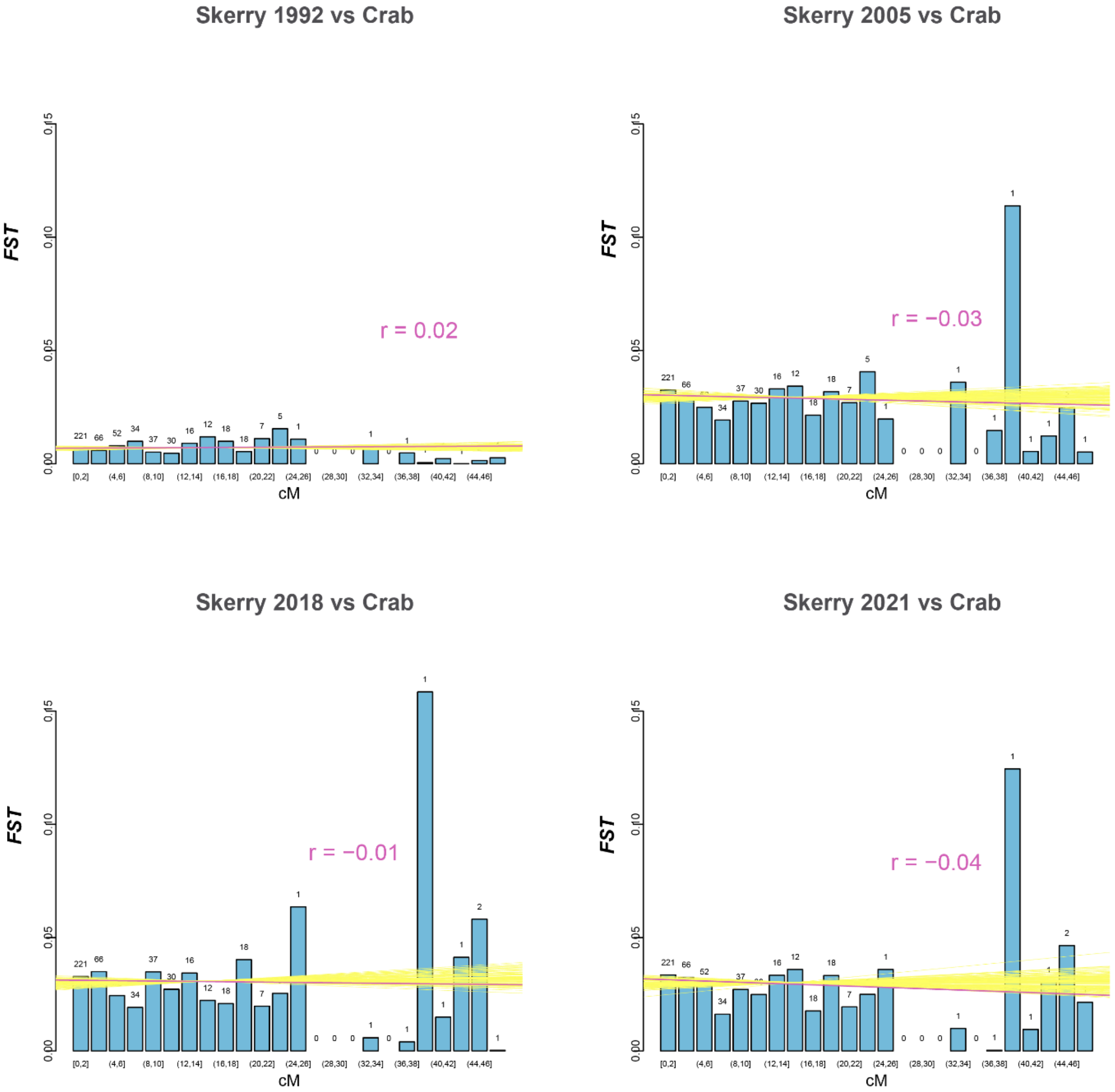

Fig. S18. Correlation between genomic distance and genetic differentiation (F_{ST}). The barcharts show the F_{ST} of control loci in the skerry samples from different years vs the combined samples from the Crab ecotype (2018+2021). The x axis is the distance in cM of each control locus to the nearest spatial outlier. The purple line is the F_{ST}-to-distance linear regression of the empirical data, accompanied by the correlation coefficient (r). In 2005, 2018, and 2021, the F_{ST} value is weakly negatively correlated to the distance to the nearest candidate outlier locus. To test whether this pattern can be generated by chance, we shifted randomly the coordinates of control loci and estimated both the linear regression and the correlation coefficient. The yellow lines are 100 simulations of F_{ST}-to-distance linear regression. The correlation observed in empirical data is always within the range of shuffled loci. Thus, there is no evidence of strong hitchhiking effects, although, this does not rule out genome-wide effects in the early generations, when selection is very strong.

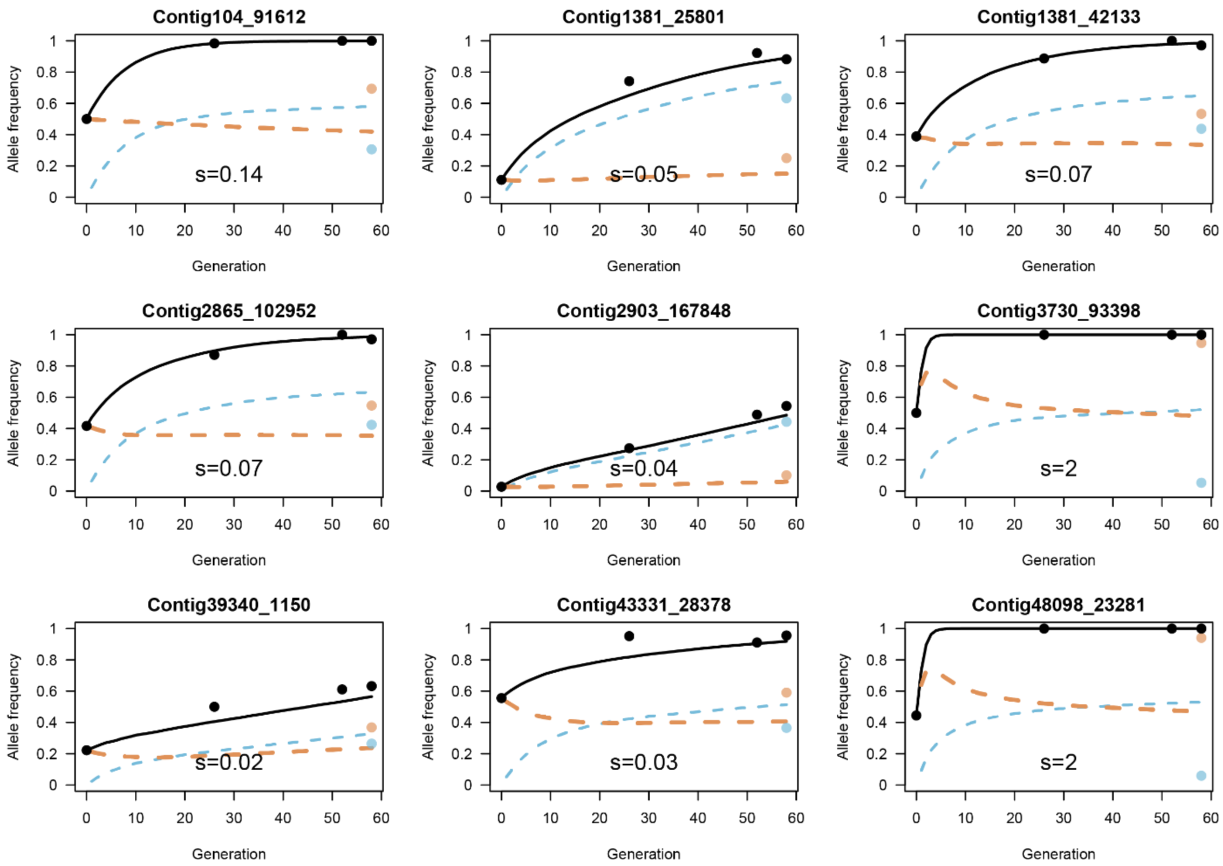

Fig. S19: **Changes over time in the frequency of the "Wave allele", i.e. the allele experiencing positive selection in Wave habitats, at loci above the expected range (spatial outliers and inversions).** Black points show the observed allele frequencies on the skerry in the four sampling generations. Lines show frequencies simulated under selection with the aim of separating the expected contribution of allelic copies descending from standing genetic variation, SGV, present in the donor population (orange lines) from those descending from variation introduced by gene flow (blue lines) (means of 1,000 replicate simulation runs). The black lines show the simulated total Wave allele frequencies (sum of SGV and gene flow). To obtain these trajectories, we first estimated the selection coefficient $\boldsymbol{s}$, using maximum-likelihood inference as described under *Demographic inference* in Materials and Methods, but fixing all demographic parameters to the values shown in Table S5. We included selection with coefficient $\boldsymbol{s}$ after gene flow in each generation (we used a haploid model, i.e. individuals with the Wave allele had a fitness of 1+s, while individuals with the Crab allele had a fitness of 1) and tested values from 0 to 2 in intervals of 0.02. We then interpolated to obtain the best value of $\boldsymbol{s}$ (shown in each plot) as described for other parameters under *Interpolation to find the truly best parameters* in Materials and Methods. We then simulated allele frequency changes at each locus under the inferred *s* and demographic model. To obtain the expected contribution of SGV and gene flow, we modelled a three-allele system: Crab allele copies (i.e. copies of the allele not favoured in Wave habitats) independent of origin ($\boldsymbol{p_C}$, not shown); Wave allele copies originating from the Crab donor ($\boldsymbol{p_{W\_SGV}}$, orange); and Wave allele copies originating from gene flow ($\boldsymbol{p_{W\_GF}}$, blue). The latter two were favoured with coefficient $\boldsymbol{s}$. In the beginning of the simulations, $\boldsymbol{p_{W\_SGV}}$ was set to the observed Wave allele frequency on the skerry in 1992 and $\boldsymbol{p_{W\_GF}}$ to 0. $\boldsymbol{p_W}$ (black) is the sum of

$\boldsymbol{p_{W\_SGV}}$ and $\boldsymbol{p_{W\_GF}}$. The orange and blue points in the final generation are the expected contributions of standing genetic variation (orange) and gene flow (blue) based on a simple analytical approximation (see *Mathematical approximation to the origin of the adapted alleles* in Supplementary Text). The approximation breaks down when the starting frequency of the Wave allele is high, violating assumptions. For two SNPs (Contig91839_37927 and Contig55826_16617), observed and simulated allele frequency changes differ strongly, indicating that a single selection coefficient cannot describe the observed allele frequency changes well. This is potentially due to the breakup of linkage disequilibrium between the SNP and directly selected loci over time. For these SNPs, the contribution of gene flow vs. standing variation is not shown. This figure continues on the next page.

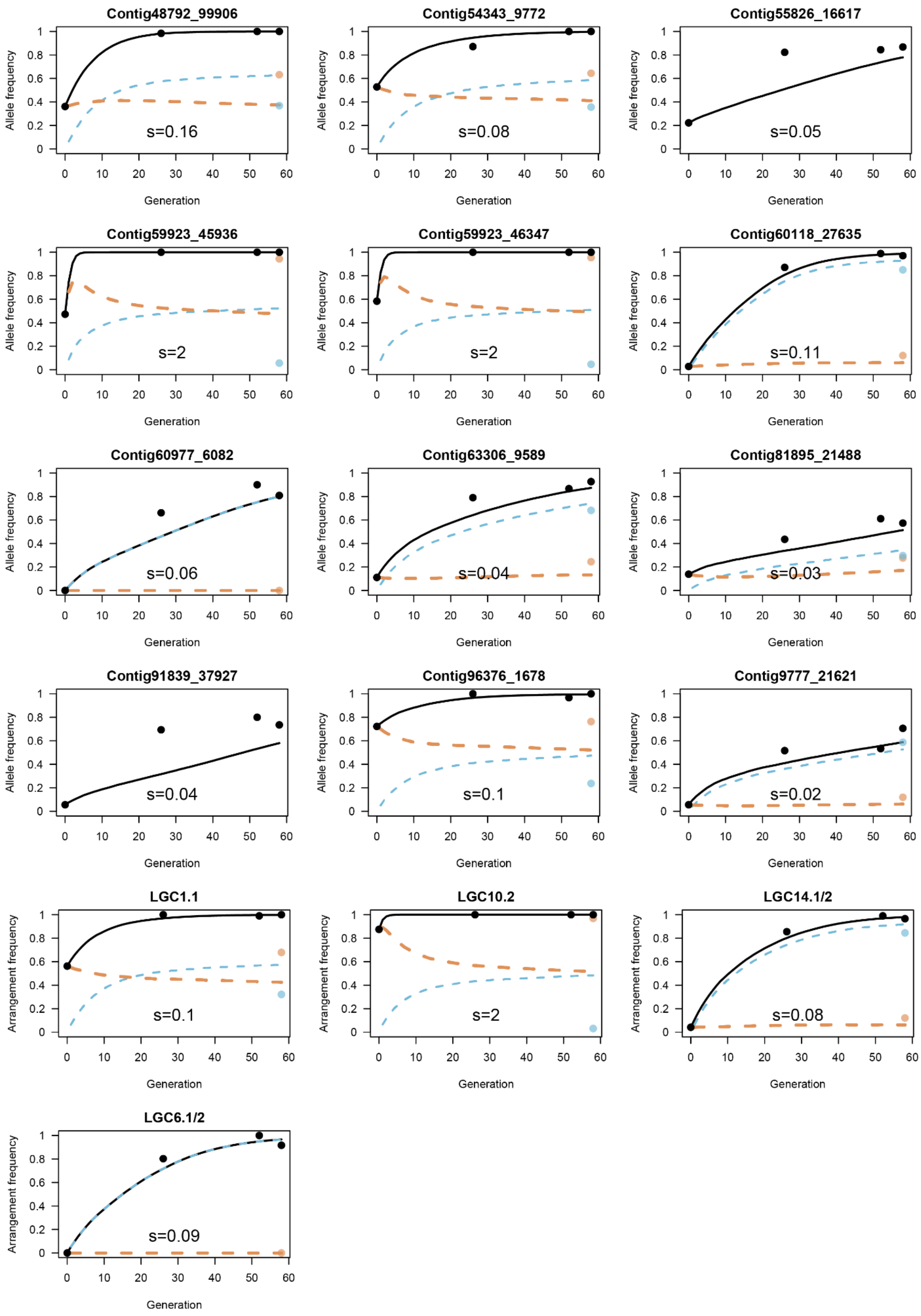
Contig48792_99906
s=0.16
Contig54343_9772
s=0.08
Contig55826_16617
s=0.05
Contig59923_45936
s=2
Contig59923_46347
s=2
Contig60118_27635
s=0.11
Contig60977_6082
s=0.06
Contig63306_9589
s=0.04
Contig81895_21488
s=0.03
Contig91839_37927
s=0.04
Contig96376_1678
s=0.1
Contig9777_21621
s=0.02
LGC1.1
s=0.1
LGC10.2
s=2
LGC14.1/2
s=0.08
LGC6.1/2
s=0.09
Allele frequency
Arrangement frequency
Generation
0
0.2
0.4
0.6
0.8
1
0
10
20
30
40
50
60

Table S1. Number of markers from each category

Marker type	Marker number
Baypass 2*	28
Baypass 3*	32
F_{ST} outlier in 1 population*	8
F_{ST} outlier in 2 population*	22
F_{ST} outlier in 3 population*	33
F_{ST} outlier in 4 population*	33
F_{ST} outlier in 5 population*	44
Clinal marker*	102
Neutral marker (control)	565
Markers in inversions	225

*Spatial outliers

Table S2. Phenotypic analysis.
Supplementary Table S2 Phenotypic Analysis.xlsx (External)

Table S3: Genotyping data used in the inference. "Usage" indicates the corresponding variable in the model and "diploid sample size" gives the number of diploid individuals to which the data set was downsampled in order to avoid missing data.

**Sample**	**Usage**	**Diploid sample size**
Crab donor 1992	k_{1992}, observed allele count on the skerry 1992	18
Skerry 2005	k_{2005}, observed allele count on the skerry 2005	31
Skerry 2018	k_{2018}, observed allele count on the skerry 2018	45
Skerry 2021	k_{2021}, observed allele count on the skerry 2021	34
Wave 2018 and 2021	Wave allele frequency p_W, source of gene flow	77

Table S4: Parameters of the demographic model.

Parameter	Description	Values
N_0	Haploid starting population size on the skerry	20, 40, 80, 160, 320
r	Growth rate in logistic population growth model	0.025, 0.05, 0.1, 0.2, 0.4
K	Haploid carrying capacity in logistic population growth model	250, 500, 1000, 2000, 4000
M	Number of haploid migrants per generation	0, 1, 2, 4, 8
f	Number of generations per year	4/3, 5/3, 2

Table S5. The mean and 95% limits of the posterior distributions, based on 500,000 random draws, using the Metropolis algorithm. The migration parameter *M* refers to haploid individuals; the diploid value would be half the shown value.

Parameter	Geometric mean	95% limits
N_0	55.4	{39.2, 79.1}
r	0.12	{0.092, 0.176}
K	1371	{950, 2168}
M	3.25	{2.77, 3.77}

Table S6: Frequencies of the Wave arrangement in the skerry and reference populations. Complex inversions (LGC6.1/2 and LGC14.1/2) have two rows in the table, corresponding to the "Wave" and the "Crab" arrangement in that order. Prefixes pC, pS, and pW account for frequency in Crab, skerry, and Wave respectively.

Inv_ID	pC1992	pS2005	pS2018	pS2021	pW2018+2021	pC2018+2021
LGC1.1	0.562	1	0.990	1	0.994	0.587
LGC1.2	0.708	0.842	0.788	0.822	0.852	0.608
LGC2.1	0.5	0.684	0.862	0.836	0.818	0.474
LGC4.1	0.791	0.868	0.892	0.914	0.918	0.757
LGC6.1/2	0	0.802	1	0.916	0.798	0
LGC6.1/2	0.708	0.144	0	0.023	0.005	0.677
LGC7.1	0.375	0.894	0.892	0.858	0.994	0.536
LGC7.2	0.708	0.894	0.921	0.934	0.947	0.607
LGC9.1	0.312	0.526	0.460	0.430	0.348	0.221
LGC10.1	0.687	0.776	0.855	0.857	0.896	0.701
LGC10.2	0.875	1	1	1	1	0.922
LGC11.1	0.833	0.907	0.99	0.945	0.979	0.779
LGC14.1/2	0.041	0.763	0.990	0.909	0.942	0
LGC14.1/2	0.666	0.092	0	0.056	0.041	0.755
LGC17.1	0	0.081	0.392	0.378	0.348	0

Table S7. The most likely combination of parameters of the demographic model from the grid. The migration parameter *M* refers to haploid individuals; the diploid value would be half the shown value.

N_0	r	K	M	f
80	0.1	2000	4	2

Table S8. Proportions of loci that fall inside the expected range of allele frequency change, above the 0.975 or below the 0.025 quantile, and above the median expected allele frequency change. The proportions of spatial outliers are based on the *full spatial outlier dataset*.

SNP category	Inside expected range	At or above 0.975 quantile	At or below 0.025 quantile	Above median
Control SNPs	0.9	0.05	0.06	0.55
Spatial outliers	0.91	0.09	0.01	0.67
Inversions	0.69	0.31	0.00	1.00

Table S9. Chi-square tests on frequencies for qualitative traits. For each qualitative trait we randomly sampled *N* phenotypes from the Crab (1992+2018+2021) and Wave (2018+2021) ecotype populations 100 times, where *N* is the number of skerry (2021) individuals with data. After each random sampling, we performed the *chi-squared* goodness-of-fit test implemented in the R package *stats* between the phenotypes of skerry and Wave and skerry and Crab samples. Finally, we averaged the statistic (X^2) and the *p*-value, and identified the different degrees of freedom, over the 100 estimates. In bold, *p*-values smaller than 0.05.

Trait	*N*	Average X^2		Degrees of freedom		Average *p*-value	
		S vs W	S vs C	S vs W	S vs C	S vs W	S vs C
Ridged	50	2.674	62.203	1	1	0.139	**6.806e-15**
Colour	46	12.967	66.546	4	4-5	**0.020**	**2.329e-12**
Patterning	44	3.271	34.119	1	1	0.107	**5.183e-09**

Table S10. Student *t*-tests on means of quantitative traits. For each quantitative trait we performed a two-sample Student's *t*-test between the samples from skerry (2021) and Wave (2018+2021) and skerry and Crab (1992+2005+2021), as implemented in the R package *stats* with default parameters. In bold, *p*-values smaller than 0.05.

	t		**Degrees of freedom**		***p*-value**	
**Trait**	**S vs W**	**S vs C**	**S vs W**	**S vs C**	**S vs W**	**S vs C**
shell_length	-0.474	-23.236	103.923	119.224	0.636	**4.317E-46**
avg_thickness	2.001	-20.347	89.244	117.978	**0.048**	**2.153E-40**
ln(gw)	-1.594	21.612	93.492	102.181	0.114	**6.710E-40**
ln(gh)	-1.334	18.198	88.406	75.304	0.185	**2.722E-29**
r0	1.132	6.210	95.525	69.327	0.260	**3.422E-08**
h0	4.055	-1.904	119.648	112.577	**8.958E-05**	0.059
a0	-1.417	14.143	88.439	74.938	0.159	**7.510E-23**
c	-1.795	-13.147	87.902	101.580	0.076	**1.182E-23**

Table S11. Fisher's exact test of independence on allele frequencies of spatial outliers beyond the expected range. For each spatial outlier beyond the expected range (from the *full spatial outlier dataset*), we performed a Fisher's exact test of independence as implemented in the R package *stats* with default parameters between the allele frequencies of skerry (2021) and combined Wave (2018+2021), and skerry and combined Crab (1992+2018+2021) samples. We created the contingency tables for each SNP based on the alternative (Alt) and Reference (Ref) allele counts. In bold, *p*-values smaller than 0.05. *N* is the number of skerry (2021) individuals.

SNP	*N*	Skerry allele counts		Wave allele counts		Crab allele counts		*p*-value	
		Alt	Ref	Alt	Ref	Alt	Ref	S vs W	S vs C
Contig55826_16617	50	84	16	73	131	55	189	**8.288e-16**	**1.818e-26**
Contig91839_37927	44	64	24	63	127	2	238	**6.897e-10**	**3.715e-45**
Contig2903_167848	41	47	35	54	134	1	241	**1.111e-05**	**6.528e-33**
Contig60118_27635	48	91	5	150	46	10	236	**5.905e-05**	**1.768e-64**
Contig60977_6082	46	74	18	116	84	0	250	**1.948e-04**	**2.995e-58**
Contig81895_21488	51	57	45	68	136	21	227	**2.045e-04**	**1.511e-20**
Contig96376_1678	48	93	3	166	34	213	33	**5.336e-04**	**0.005**
Contig39340_1150	45	35	55	104	64	181	59	**6.183e-04**	**1.171e-09**
Contig43331_28378	52	99	5	178	28	145	105	**0.018**	**8.382e-14**
Contig1381_25801	51	91	11	156	32	58	186	0.169	**6.730e-31**
Contig104_91612	49	0	98	5	199	103	149	0.178	**1.373e-18**
Contig2865_102952	43	84	2	185	1	138	114	0.235	**4.284e-16**
Contig1381_42133	48	94	2	201	1	125	123	0.243	**3.275e-20**
Contig9777_21621	49	68	30	127	75	24	222	0.302	**6.642e-28**
Contig48098_23281	49	98	0	198	4	112	140	0.307	**1.110e-27**
Contig63306_9589	45	10	80	29	163	176	66	0.460	**3.913e-25**
Contig48792_99906	47	94	0	195	3	106	136	0.553	**7.257e-27**
Contig3730_93398	49	98	0	198	2	145	103	1	**5.364e-19**
Contig54343_9772	50	0	100	1	201	114	134	1	**7.876e-22**
Contig59923_45936	50	100	0	199	1	135	113	1	**2.063e-21**

Contig59923_46347	37	74	0	181	1	132	110	1	**6.953e-17**

Table S12. Fisher's exact test of independence on arrangement frequencies of simple and complex inversions. We performed a Fisher's exact test of independence as implemented in the R package *stats* with default parameters between the inversion arrangement frequencies of skerry (2021) and combined Wave (2018+2021), and skerry and combined Crab (1992+2018+2021) samples. We created the contingency tables for each inversion based on counts of two arrangements (A and B) in simple inversions, and three arrangements (A, B and C) in complex inversions. In bold, *p*-values smaller than 0.05. *N* is the number of skerry (2021) individuals for a given inversion.

**Inversion**	***N***	**Skerry arrangement counts**			**Wave arrangement counts**			**Crab arrangement counts**			***p*-value**	
		**A**	**B**	**C**	**A**	**B**	**C**	**A**	**B**	**C**	**S vs W**	**S vs C**
LGC7.1	39	11	67	-	1	187	-	120	122	-	**7.39E-06**	**1.28E-08**
LGC6.1/2	42	0	80	14	10	182	54	240	22	108	**0.027**	**4.48E-60**
LGC11.1	46	87	5	-	193	3	-	185	49	-	0.115	**4.24E-04**
LGC14.1/2	44	88	2	4	192	0	6	8	102	230	0.148	**2.51E-74**
LGC9.1	43	49	37	-	125	67	-	184	58	-	0.227	**0.001**
LGC10.1	49	85	13	-	183	19	-	169	73	-	0.323	**9.28E-04**
LGC1.2	48	79	17	-	174	30	-	152	90	-	0.500	**4.43E-04**
LGC17.1	41	51	31	-	129	69	-	244	0	-	0.681	**1.65E-21**
LGC7.2	46	6	86	-	10	182	-	91	153	-	0.783	**2.57E-09**
LGC2.1	49	82	16	-	163	35	-	116	126	-	0.870	**6.15E-10**
LGC1.1	49	0	98	-	1	197	-	101	141	-	1	**4.67E-19**
LGC4.1	47	86	8	-	180	16	-	185	57	-	1	**0.001**
LGC10.2	49	98	0	-	202	0	-	221	21	-	1	**8.37E-04**

Table S13. Changes in phenotypes expressed as Haldanes or as Darwin numerators for comparison with rates of change in other systems. Trait *r0* was excluded because all change was plastic and changes in other traits were calculated after the plastic change estimated from the model of selection with one generation per year and the overall estimate of genetic variance. The phenotypic standard deviation was calculated from the average of the variance at each sample point. Change in $Haldanes = (X_2/SD(X) - X_1/SD(X))/G$ and the $Darwin\ numerator = log(X_2) - log(X_1)$, where X_1 is the mean phenotype at the start and X_2 is the mean phenotype at the end of the time interval, $SD(X)$ is the phenotypic standard deviation and G is the duration of the interval in generations. Signs in the table for Haldanes and the Darwin numerator indicate the direction of evolution.

	**Trait**				
	**log(shellLength)**	**gw**	**log(gh)**	**a0**	**c**
phenotypic SD	0.193	0.010	0.086	0.025	0.105
plastic effect	-0.114	0.010	0.201	0.023	-0.088
Sample means:					
donor	2.189	0.092	-2.232	0.284	1.389
skerry96	1.862	0.128	-1.961	0.333	1.293
skerry02	1.907	0.128	-1.916	0.346	1.212
skerry05	1.536	0.138	-1.895	0.359	1.198
skerry18	1.644	0.133	-1.913	0.357	1.267
skerry21	1.457	0.125	-1.935	0.348	1.145
wave	1.477	0.128	-1.912	0.355	1.177
**Rate of change in Haldanes (assuming 1 generation per year):**					
donor to 96	-0.423	0.888	0.785	0.494	-0.228
donor to 02	-0.146	0.355	0.366	0.247	-0.168
donor to 05	-0.260	0.348	0.300	0.230	-0.140
donor to 18	-0.109	0.153	0.142	0.113	-0.045
donor to 21	-0.131	0.113	0.119	0.089	-0.080
**Rate of change in Haldanes (assuming 2 generation per year):**					
donor to 96	-0.212	0.444	0.392	0.247	-0.114
donor to 02	-0.073	0.178	0.183	0.123	-0.084
donor to 05	-0.130	0.174	0.150	0.115	-0.070
donor to 18	-0.054	0.076	0.071	0.057	-0.022
donor to 21	-0.065	0.056	0.059	0.044	-0.040
**Darwin numerator:**					
donor to 96	-0.108	0.236	-0.035	0.082	-0.006
donor to 02	-0.085	0.236	-0.058	0.119	-0.070
donor to 05	-0.301	0.311	-0.069	0.156	-0.082
donor to 18	-0.233	0.269	-0.060	0.152	-0.026

donor to 21	-0.354	0.213	-0.048	0.127	-0.127

Code S1. Mathematica Workbook: Skerry interpolation 9.23 v2.nb. This notebook takes a list of parameter combinations and their negative log likelihoods as described under *Demographic inference* in Materials and Methods, to interpolate the likelihood surface. It then uses the Metropolis algorithm to sample parameter sets in proportion to their likelihood. This file has a .nb format, a type of structured document created by Wolfram Research Mathematica and can be accessed using the free Wolfram Mathematica player, and run using Mathematica. More information about the .nb format on https://www.wolfram.com/technologies/nb/. The Skerry interpolation 9.23 v2.nb file is provided separately. A PDF version of this notebook is provided at the end of the Supplementary Materials.

PDF Version of the Mathematica Workbook *Skerry interpolation 9.23 v2.nb*

This notebook takes a list of parameters & their negative log likelihoods, and interpolates to give a smooth sampling. It then uses the Metropolis algorithm to sample parameter sets in proportion to their likelihood.

SetDirectory to point to the data file, and Evaluate Initialisation

The data

The data are supplied as a list of the form {N_0, r, K, M, T, −log(L)}

In[ ]:=
```
data[[1]]
```
Out[ ]=
```
{20, 0.025, 250, 0, 1.33333, 7018.16}
```

These are the parameter values supplied:

In[ ]:=
```
Union /@ Drop[Transpose[data], -1]
```
Out[ ]=
```
{{20, 40, 80, 160, 320}, {0.025, 0.05, 0.1, 0.2, 0.4},
 {250, 500, 1000, 2000, 4000}, {0, 1, 2, 4, 8}, {1.33333, 1.66667, 2}}
```

Interpolation on a log scale

The parameter grid is evenly spaced on a log scale, and so the interpolation is also on a log scale. (Interpolating on the original scale causes problems, because the parameters are then unevenly spaced). The interpolation passes through every data point, and fills in-between using a cubic curve.   Because M=0 is included, the transformation is lM = log[M + 0.5], M = Exp[lM] − 0.5

Note: this uses natural logs

This transforms to a (natural) log scale, and interpolates. The error message arises because there are only three generation times, and so thhe interpolation uses a quadratic rather than a cubic.

In[15]:=
```
intB = Interpolation[
  data /. {n_, r_, k_, M_, t_, L_} :> {Log[n], Log[r], Log[k], Log[M + 0.5], t, L}]
```

Interpolation : Requested order is too high; order has been reduced to {3, 3, 3, 3, 2 }.

Out[15]=
```
InterpolatingFunction[
  Domain: {{3., 5.77 }, {−3.69, −0.916 }, {5.52, 8.29 }, {−0.693, 2.14 }, {1.33, 2. }}
  Output: scalar
]
```

This fixes M=4, T=2 and finds the MLE for {log[N_0], log[r], log[K]}, starting the search somewhere plausible {80, 0.1, K=800}, and setting bounds on the search domain:

In[29]:=

```
fmb =
 FindMinimum[intB[ln, lr, lk, Log[4 + 0.5], 2], {ln, Log[80], Log[20], Log[320]},
  {lr, Log[0.1], Log[0.025], Log[0.4]}, {lk, Log[800], Log[250], Log[4000]}]
```

FindMinimum : The line search decreased the step size to within the tolerance specified by AccuracyGoal and PrecisionGoal but was unable to find a sufficient decrease in the function. You may need more than MachinePrecision digits of working precision to meet these tolerances.

Out[29]=

```
{5666.39, {ln → 4.08265, lr → -2.12234, lk → 7.23995}}
```

This also allows M to vary, which slightly increases the likelihood:

In[30]:=

```
fmb1 = FindMinimum[intB[ln, lr, lk, lm, 2],
  {ln, Log[80], Log[20], Log[320]}, {lr, Log[0.1], Log[0.025], Log[0.4]},
  {lk, Log[800], Log[250], Log[4000]}, {lm, Log[4 + 0.5], Log[0.5], Log[8 + 0.5]}]
```

FindMinimum : The line search decreased the step size to within the tolerance specified by AccuracyGoal and PrecisionGoal but was unable to find a sufficient decrease in the function. You may need more than MachinePrecision digits of working precision to meet these tolerances.

Out[30]=

```
{5662.62, {ln → 3.9973, lr → -2.0709, lk → 7.19375, lm → 1.32586}}
```

These are the MLE on the original scale, allowing M to vary, or fixing it at 4:

In[ ]:=

```
Exp[{ln, lr, lk, lm} /. fmb1[[2]]] + {0, 0, 0, 0.5}
```

Out[ ]=

```
{54.4509, 0.126072, 1331.09, 4.26541}
```

In[ ]:=

```
Exp[{ln, lr, lk} /. fmb[[2]]]
```

Out[ ]=

```
{59.3027, 0.119751, 1394.02}
```

The minimum in the data is only slightly higher than the MLE, which seems reasonable:

In[ ]:=

```
intB[Log[80], Log[0.1], Log[2000], Log[4 + 0.5], 2]
```

Out[ ]=

```
5669.36
```

The left plot fixes T=2, M=3.26, r=0.1202, the right plot fixes $N_0 = 55.6$, $K = 1335$. Contours are spaced at unit intervals. For 2 degrees of freedom, a loss of log(L) of 3 corresponds to $\chi_2^2 = 6$, or P=5%; thus, three contours down give the 3-unit support limits, corresponding to 95% confidence intervals.

Out[240]=

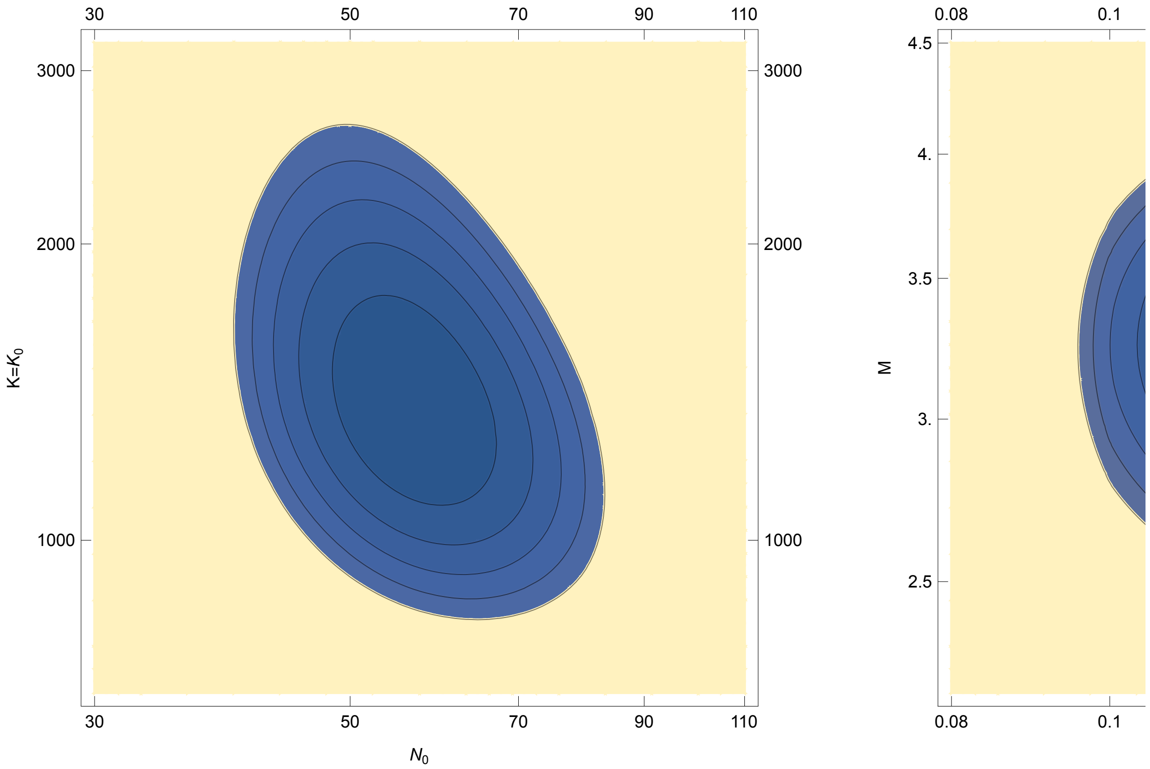

Optimising N_0, r for given K

If we optimise N_0 and r for given K, we get a smooth function with a definite minimum

In[ ]:=
```
fmtb = Table[Prepend[FindMinimum[intB[ln, lr, lk, Log[4 + 0.5], 2],
    {ln, Log[60], Log[20], Log[320]}, {lr, Log[0.12], Log[0.025], Log[0.4]}],
   Exp[lk]], {lk, Log[250.], Log[4000], Log[4000/250]/10}];
TableForm[fmtb, TableDepth → 2]
```

Out[ ]//TableForm=

250.	5757.92	{ln → 5.26271, lr → -0.916291}
329.877	5722.78	{ln → 4.2954, lr → -1.75283}
435.275	5699.43	{ln → 4.12883, lr → -1.76095}
574.349	5683.25	{ln → 4.04163, lr → -1.80412}
757.858	5673.21	{ln → 4.00897, lr → -1.875}
1000.	5668.08	{ln → 4.01932, lr → -1.97126}
1319.51	5666.43	{ln → 4.07061, lr → -2.09685}
1741.1	5666.89	{ln → 4.13516, lr → -2.22506}
2297.4	5668.36	{ln → 4.15695, lr → -2.30259}
3031.43	5670.36	{ln → 4.10421, lr → -2.30259}
4000.	5672.12	{ln → 4.05919, lr → -2.30259}

In[]:=

```
ListLinePlot[fmtb /. {k_, l_, rl_List} :→ {k, l}]
```

Out[]=

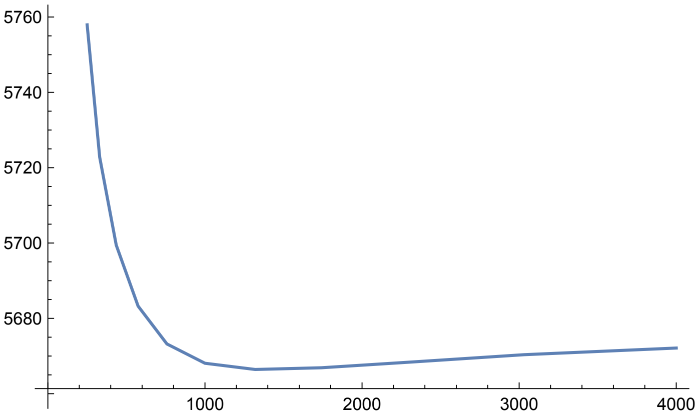

MLE for three values of T

Note: this uses natural logs

These are -log(L) and the MLE for the three values of T. T=2 seems most likely; the choice of T makes little difference to the estimates.

In[]:=

```
TableForm[Prepend[
  {tvalues[[#]], fm[#][[1]], Exp[ln], Exp[lr], Exp[lk], Exp[lm] - 0.5} /. fm[#][[2]] & /@
   {1, 2, 3},
  {"T", "-log(L)", "N0", "r", "K", "M"}]]
```

Out[]//TableForm=

T	-log(L)	N_0	r	K	M
1.33333	5665.23	55.4199	0.102179	4000.	3.23059
1.66667	5663.39	55.5942	0.120153	1335.05	3.26502
2	5662.62	54.4509	0.126072	1331.09	3.26541

Generating 5×10^5 random values, for T=2; burn-in of 10^4

In[106]:=

```
burn = 10^4; run = 5 × 10^5;
Timing[xl = Drop[randomWalk[burn + run, 1.05, 3], burn];]
```

Out[107]=

```
{483.009, Null}
```

The file contains 5×10^5 values of $\{N_0, r, K, M, -\log(L)\}$:

In[108]:=

```
Export["random values 23 Sept 2023.csv", Flatten /@ xl];
```

In[109]:=

```
ListLinePlot[Mean /@ Partition[Last /@ xl, 1000]]
```

Out[109]=

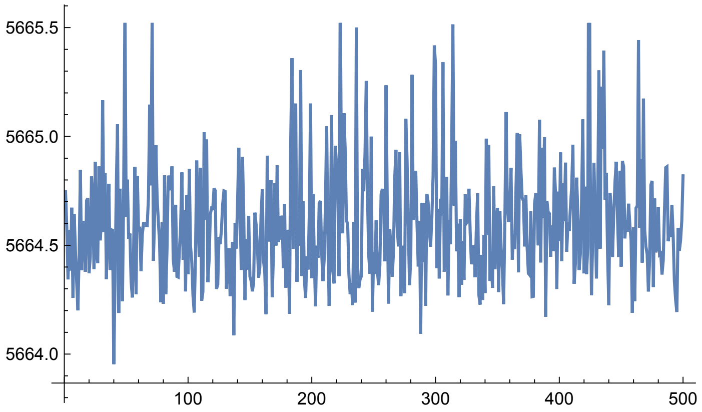

Distribution of the parameters (T=2)

Posterior distribution (points) superimposed on contours of log likelihood (spacing 1). Left: N_0 vs K. right: r vs. M . Contours on the left plot fixes T=2, M=3.26, r=0.1202; the right plot fixes $N_0 = 55.6$, $K = 1335$. Note that these distributions are not quite the same: the posterior distribution averages over the posterior distribution of the other two parameters, whereas the contours show the log likelihood with the other two parameters fixed at their MLE.

Out[214]=

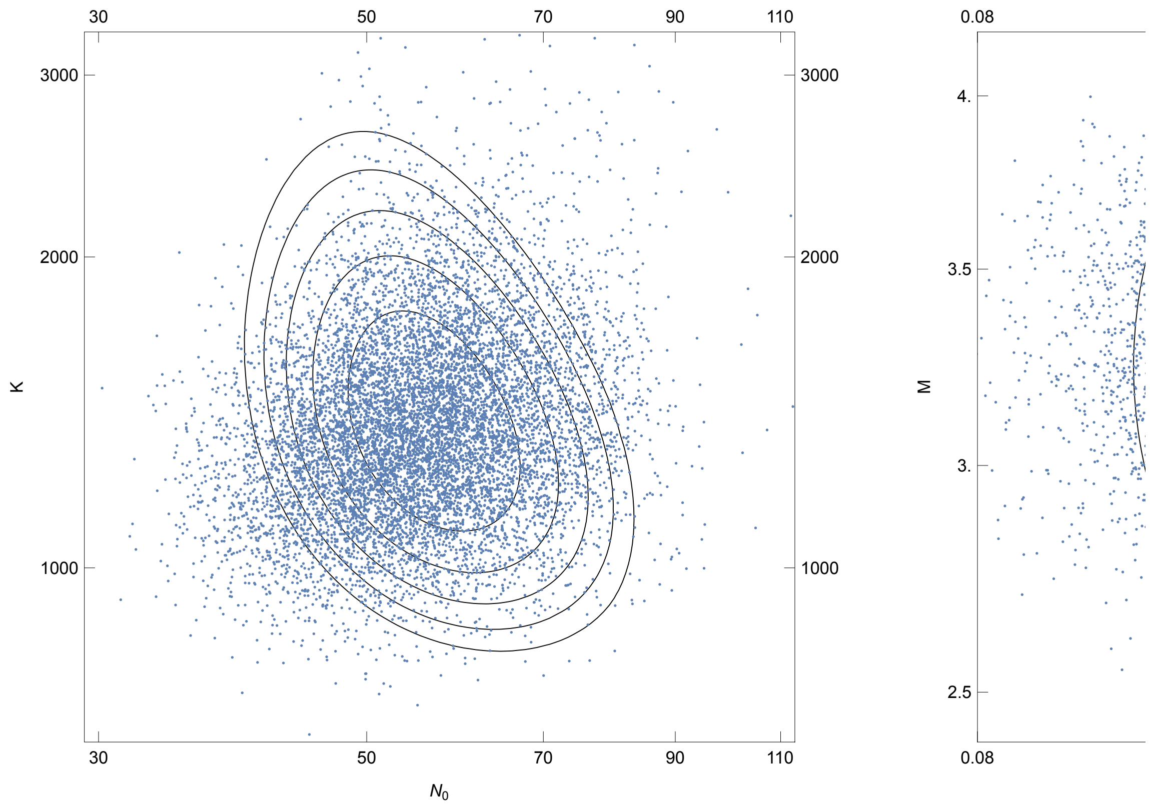

These are the posterior distributions of the 4 parameters, for T=2:

Out[135]=

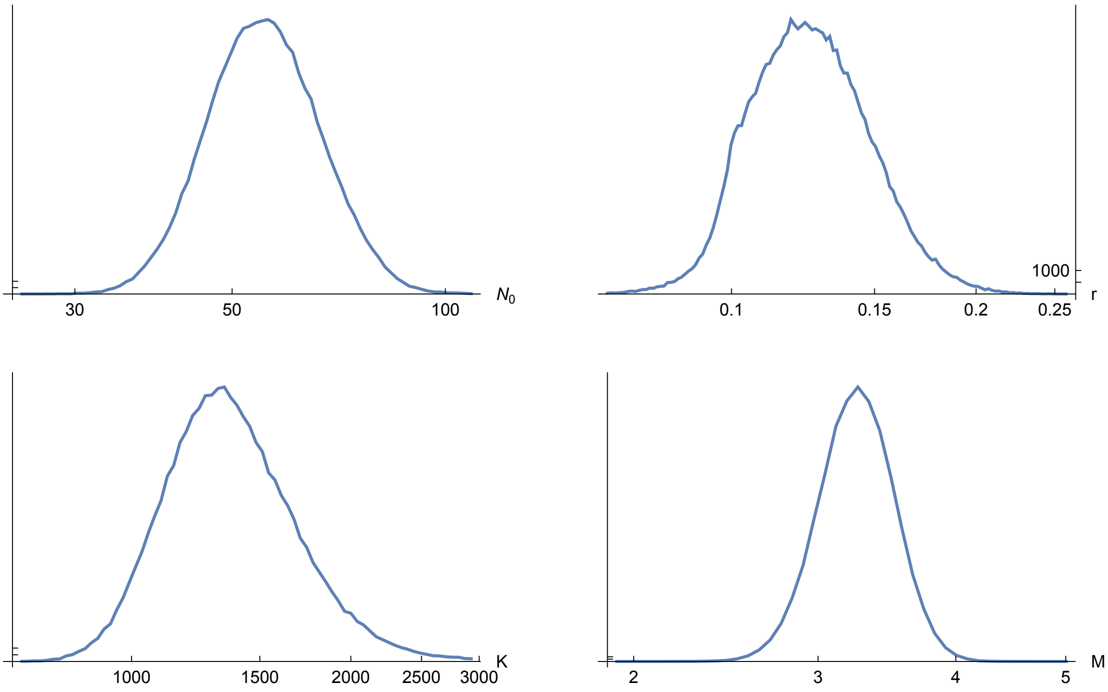

These are the mean and the 95% limits of the posterior distribution:

Out[136]//TableForm=

	mean	95% limits
N_0	55.3948	{39.1577, 79.1246}
r	0.124778	{0.0922092, 0.175655}
K	1370.91	{949.594, 2168.92}
M	3.25047	{2.76906, 3.7712}

Some checks

The mean of the random walk (top) is close to the MLE (bottom)

Out[138]//TableForm=

N_0	r	K	M
55.3948	0.124778	1370.91	3.25047
55.5942	0.120153	1335.05	3.26502

The minimum -log(L) achieved in the random walk (top) is slightly better than the estimated MLE from interpolation. -2log(L) should follow a χ_4^2 distribution. The mean -2log(L) in the random walk is 3.97 above this, about the same as the predicted 4 (the # of parameters), and the variance is also close to the predicted 8

Out[140]//TableForm=

5662.62	3.97153	8.24493
5663.39	4	8

In[141]:=

```
Histogram[xl[[All, -1]]]
```

Out[141]=

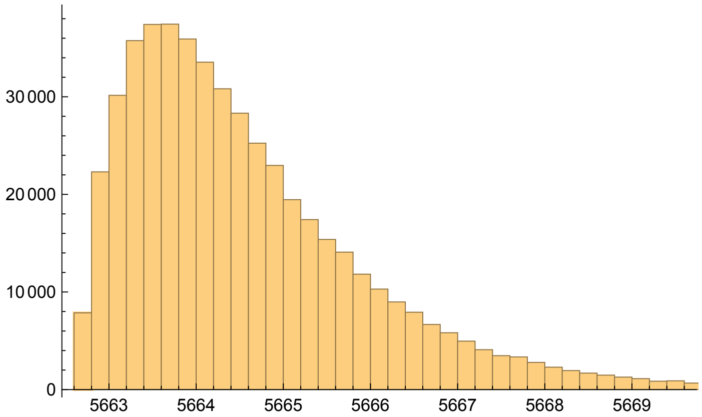

There is no suggestion of a systematic change in -log(L) over the random walk. This plots the means of every 1000 points:

In[144]:=

```
ListLinePlot[Mean /@ Partition[xl[[All, -1]], 1000]]
```

Out[144]=

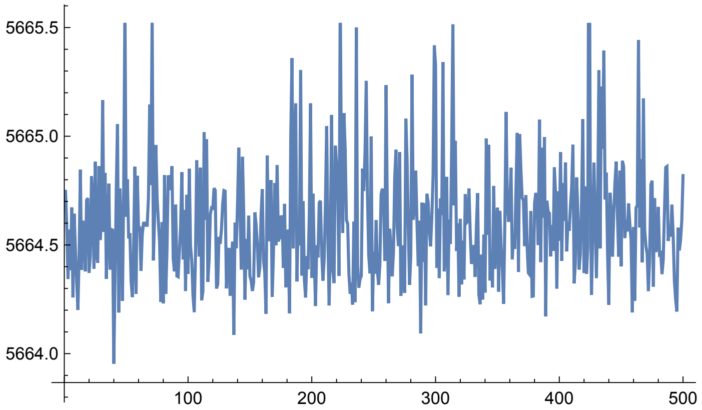

Definitions

In[1]:=

```
SetDirectory["/Users/NickBarton/Manuscripts/Skerries/"];
(* set this directory to point to the data file *)
data::usage =
  "data stores an array where each row gives the 5 parameters, followed by
    the negative log likelihood, in the form {N0,r,K,M,T,-log(L)}";
data = Flatten[
   Map[StringSplit, Import["SEQSNPTM004_RESULTS Aug23.txt", "CSV"], {2}], 1];
data = Map[ToExpression, Drop[data, 1], {2}];
```

In[5]:=

```
tvalues::usage = "tvalues lists the three values of t";
tvalues = Union[data[[All, 5]]];
```

In[7]:=

```
intC::usage =
  "intC[t] stores an interpolation on log[N_0],log[r],log[K],log[M+0.5],
    given t (which must be one tvalues)";
intC[t_] := intC[t] =
   Interpolation[Cases[data, {_, _, _, _, t, _}] /.
     {n_, r_, k_, M_, _, L_} :> {Log[n], Log[r], Log[k], Log[0.5 + M], L}];
```

```
intD::usage =
  "intD[t] gives an interpolation on log[N_0],log[r],log[K],log[M+0.5], given
    t (which must be one tvalues). Returns ∞ if the arguments lie
    outside the range of the interpolation, as given by intC[t][[1]].";
intD[t_][x_List] := If[withinQ[intC[t]⟦1⟧][x], intC[t] @@ x, ∞];
```

In[71]:=

```
withinQ::usage = "withinQ[{{x_{1,0},x_{1,1}},…}][{x_1,…}]
    checks whether the point is within a rectabgular domain";
withinQ[xl_List][x_List] := And @@ MapThread[#2⟦1⟧ ≤ #1 ≤ #2⟦2⟧ &, {x, xl}];
```

In[9]:=

```
fm::usage =
  "fm[j] gives minimises wrt log[N_0],log[r],log[K],log[M+0.5]; j=1,2,3
    corresponds to t=1.333, 1.667, 2";
fm[j_] := fm[j] = Module[{t = Union[data⟦All, 5⟧]⟦j⟧},
    FindMinimum[intC[t][ln, lr, lk, lm], {ln, Log[100], Log[20], Log[320]},
     {lr, Log[0.1], Log[0.025], Log[0.4]}, {lk, Log[2000], Log[250], Log[4000]},
     {lm, Log[4 + 0.5], Log[0 + 0.5], Log[8 + 0.5]}]];
```

In[87]:=

```
randomWalk::usage =
  "randomWalk[n,λ,j] makes n random draws from the posterior distribution;
    j=1,2,3 corresponds to the three values of T.  If a trial is
    rejected, the step is decreased by a factor λ, and vice versa.";
randomWalk[n_Integer, λ_, j : (1 | 2 | 3)] :=
  Module[{δ = 0.1, x, L, xL, L1, dx, τ = tvalues⟦j⟧},
   x = Mean /@ intC[τ]⟦1⟧; L = intD[τ][x]; xL = {{x, L}};
   Do[dx = δ RandomReal[{-1, 1}, 4];
    L1 = intD[τ][x + dx];
    If[Exp[L - L1] > Random[],
     x = x + dx; δ = δ * λ; L = L1,
     δ = δ / λ];
    AppendTo[xL, {x, L}], {n}];
   xL];
```

In[13]:=

```
limits::usage = "limits[{x_1,…},P] gives the parameter range
    that includes a fraction 1-P, with P/2 on each side.";
limits[x_List, p_] := Module[{k = Round[Length[x] (p / 2)]}, Sort[x]⟦{k, -k}⟧];
```
